# Vegetation change detection and recovery assessment based on post-fire satellite imagery using deep learning

**DOI:** 10.1038/s41598-024-63047-2

**Published:** 2024-06-01

**Authors:** R. Shanmuga Priya, K. Vani

**Affiliations:** Information Science and Technology, College of Engineering Guindy, Anna University, 12 Sardar Patel Road, Chennai, 600 025 India

**Keywords:** Wildfire, Change detection, Ensemble learning, Deep learning, Environmental sciences, Natural hazards, Fire ecology, Forestry, Restoration ecology

## Abstract

Wildfires are uncontrolled fires fuelled by dry conditions, high winds, and flammable materials that profoundly impact vegetation, leading to significant consequences including noteworthy changes to ecosystems. In this study, we provide a novel methodology to understand and evaluate post-fire effects on vegetation. In regions affected by wildfires, earth-observation data from various satellite sources can be vital in monitoring vegetation and assessing its impact. These effects can be understood by detecting vegetation change over the years using a novel unsupervised method termed Deep Embedded Clustering (DEC), which enables us to classify regions based on whether there has been a change in vegetation after the fire. Our model achieves an impressive accuracy of 96.17%. Appropriate vegetation indices can be used to evaluate the evolution of vegetation patterns over the years; for this study, we utilized Enhanced Vegetation Index (EVI) based trend analysis showing the greening fraction, which ranges from 0.1 to 22.4 km^2^ while the browning fraction ranges from 0.1 to 18.1 km^2^ over the years. Vegetation recovery maps can be created to assess re-vegetation in regions affected by the fire, which is performed via a deep learning-based unsupervised method, Adaptive Generative Adversarial Neural Network Model (AdaptiGAN) on post-fire data collected from various regions affected by wildfire with a training error of 0.075 proving its capability. Based on the results obtained from the study, our approach tends to have notable merits when compared to pre-existing works.

## Introduction

Wildfires, a significant natural disaster, can cause changes to compositions and lead to ecological imbalance, along with substantial modifications to the ecosystem of a region. Identifying and monitoring land cover changes are necessary for managing a balanced ecosystem and economic studies at various levels^1^. Forest cover change identification is significant for continuous environmental monitoring and thoroughly examining urgent ecological issues, including degradation of natural resources, loss in biodiversity, and forest cover loss^2^. For environmental research as well as forest monitoring and management, it’s essential to comprehend how fire affects forests and to identify the patterns of time and space in the recovery of forests after fire^3–6^.

Traditional approaches including ground surveys are costly and require much time. This can be attributed to various factors that make understanding the changes made to the vegetation practically infeasible. To overcome this using remote sensing, one can monitor changes across broad areas economically and effectively^7^. Change detection can provide crucial data for handling disasters, policy creation, adequate land cover and forest management^8^. Unprecedented fire seasons worldwide prompt a need to understand how vegetation responds to evolving fire patterns. Vegetation Indices are used to asses and analyse the regrowth of vegetation in a region after any major disaster, and they are a technique regularly used by researchers for this purpose. Data collected at three different post-fire periods revealed distinctions between vegetation in a control forest and a regenerating region^9^. Due to changes in land use and climate, wildfires have become more frequent and severe. Many tend to last longer, which causes a lot of damage to vegetation resulting in a need to quantify vegetation recovery in fire-affected regions so that appropriate steps can be taken to speed up this process. Assessing vegetation recovery poses challenges because of the extended duration required to capture the unique traits of an ecosystem. Time-series-based remote sensing data allows for assessments made before, during and after the fire and enhances accuracy.

## Related works

Accurate and effective methods for collecting quantitative forest change data from remotely sensed photos, developed and tested in a monitoring-based study, are essential for the ongoing monitoring of forest clearing in the MBR^10^. This change detection analysis is a valuable method for characterizing the alterations noticed throughout every category of land utilization. High resolution satellite data would properly enhance the classification of land use.. The normalized difference vegetation index technique has been utilized for feature extraction with a range of threshold values. NDVI approach yields more effective outcomes for vegetation with different types and dispersed vegetation from a multispectral remote sensing picture^11^. Satellite data provides unparalleled effectiveness in tracking and measuring extensive changes in landscape across time. However, radiometric consistency across multi-temporal imagery must be ensured to detect changes in terrain clearly^12^. There is a need to integrate both deep learning techniques and Object-based image analysis techniques for change detection, making this process much more efficient and accurate^13^. Various approaches have been utilized for changed area detection, which includes Patch-Based detection^14^, Pixel-Based to Object-Based approaches^15^, and Landsat time series data^16^. The accuracy of various object-based detection techniques depends on image segmentation quality and many approaches struggle with under-segmentation errors^17^. This can be due to the sparse nature of the features present in images.

Enhancing our understanding of how ecosystem imbalance and changes in the order of fires due to climate can influence post-fire development speed and adaptability is crucial for improving predictions of community responses to fire in the context of climate change^18^. When photosynthetic vegetation is destroyed by fire, reflectance in the visible to near-infrared range drastically decreases. Meanwhile, increased ash increases reflectance in the short and middle infrared range^19,20^. Due to their ability to absorb residual approximations, they can provide a standardized representation of vegetation or something close to it, which improves monitoring to a more significant extent^21–23^. Vegetation Indices are very useful and are compliant when the region for monitoring is vast^24,25^. Since satellite data has become accessible daily, it becomes feasible to efficiently track vegetation regrowth more granularly using the Normalized Difference Vegetation Index (NDVI)^26^. Studies indicate that due to recurrent fires in a region, another species replaces a vegetation species and this process can be quite prolonged in nature owing to the nature of both species^27^. This should be considered while evaluating the possibility of vegetation regrowth, which can be attributed to factors like soil pH, nitrogen content, etc. VIIRS proved an excellent way to estimate spectral indices over Landsat, given the decommissioning of the MODIS satellites^28^. While previous studies have commonly employed NBR with NDVI for extracting regions of plant regeneration, these approaches are susceptible to atmospheric effects and soil brightness^29^.

Spectral indices, including NDVI, EVI and distinction in various indices spanning the years before and after the fire, can be calculated and analysed^30^. NDVI proved more effective in tracking vegetation changes^31^. To analyse post-fire vegetation recovery, using multi-wavelength satellite data is an approach that utilizes various remotely sensed parameters, including visible–infrared vegetation indices^32^. The vegetation index credibility for effective vegetation monitoring can be further attributed to its use for drought monitoring^33^. Given the various applications utilizing NDVI derived from MODIS and VIIRS-based products, we can see that MODIS and VIIRS NDVI data are interchangeable for applications with an uncertainty ranging from 0.02 to 0.05, depending on the scale of spatial aggregation, typically consistent with the individual datasets uncertainty making data provided by the VIIRS-based products especially the NDVI data good enough to perform monitoring^34^. Even though vegetation indices can give insight into vegetation recovery in a region, concatenating this along with an unsupervised learning approach has not been explored a lot before and can provide more accurate information about that region. STAnet is a computational model designed to detect auditory spatial attention from EEG signals by integrating spatial and temporal data. It dynamically assigns weights to EEG channels through spatial and temporal attention mechanism and focuses on temporal patterns in EEG signals. Tested on ASAD datasets, STAnet outperforms other models significantly under various conditions, even with a 1-s decision window compared to the typical 10-s requirement. It maintains competitive performance with varying EEG signal densities, from 64 to as few as 16 channels. This study suggests the feasibility of efficient online decoding of low-density EEG signals, promising practical applications in human–computer interfaces and aiding hard-of-hearing individuals. STAnet's development represents a significant advancement toward implementing ASAD in real-world scenarios, with implications for neuroscience, human–computer interaction, and assistive technology^35^. After obtaining high-level features through SFA and the Bi-Attention Mechanism, the slow feature analysis model is applied to separate changed pixels from unchanged ones. Features that change slowly indicate unchanged pixels, representing underlying structures or patterns that remain consistent over time. Finally, a threshold method is used to generate a change map, delineating regions of significant change within the remote sensing images^36^, providing helpful information that led to the development of our approach.

In summary, we can come to an understanding that Sparse Autoencoder performs better than simple Autoencoder to extract features, which can significantly improve the change detection process when it is used along with Deep Embedded Clustering, typically used for segmentation purposes due to the better performance of the algorithm and its relatively simple implementation. Performing vegetation inference through the usage of Sen’s slope based on EVI trend analysis along with Ensemble learning can be used to identify precisely whether there is a possibility for vegetation regrowth. GANs are of various types which depend on the task they tend to perform, and taking this as inspiration we propose a deep learning-based unsupervised adversarial network. AdaptiGAN, trained on the EVIIRS NDVI composites obtained after several pre-processing steps, can provide a more efficient and accurate description of the level of vegetation recovery in the fire-affected regions.

## Data and methodology

### Data and preprocessing

A dataset comprising of pre-fire and post-fire satellite images was constructed to perform vegetation change detection analysis after the fire. This dataset comprised 3600 pre-fire and post-fire Normalized Difference Vegetation Index (NDVI) images of the EROS Moderate Resolution Imaging Spectroradiometer (eMODIS) collection which is obtained from the U.S. Geological Survey (USGS) website (https://earthexplorer.usgs.gov/) for regions which were affected by fire and the search criteria for this was based upon the periods for pre-fire and post-fire images. Appropriate corrections were made to ensure consistency and accuracy. The QGIS Software calculates the NDVI index and splits the region into several classes of vegetation based on the index value of each pixel of the multi spectral image.

Remote sensing data is collected yearly from 2000 to 2022 for vegetation regrowth assessment. The MODIS 250 m/pixel 16-day composite vegetation indices dataset from the World Database on Protected Areas (WDPA) dataset is used for this purpose. From the obtained composites, an image-based dataset was constructed. Additionally, predicting whether vegetation regrowth is possible in a region involves collecting soil data from the soil grid database. This data consists of columns including Location Co-ordinates, soil pH, nitrogen content, organic carbon content, bulk density and soil groups. All these attributes are then concatenated and represented as a single dataset. SoilGrids is a comprehensive system for automated mapping of soil properties globally, utilizing machine learning algorithms and a vast database of soil profiles and covariate data. It offers updatable soil property and class maps 1 km and 250 m at two spatial resolutions. These maps include predictions of various site characteristics, such as depth to bedrock, physical and chemical soil properties like bulk density, clay, silt, sand percentages, and more, at depths up to 2 m. Additionally, SoilGrids provides soil classifications, including the most probable class and predicted probabilities for each WRB soil unit and Soil Taxonomy suborder. While the mapping accuracy ranges from 30 to 70% for different properties, SoilGrids offers an objective estimate of mapping uncertainty. This allows users to assess the impact of prediction uncertainty on scenario or model testing. Initially developed using national and regional soil profile databases, SoilGrids encourages contributions from organizations or individuals to improve local predictions by providing additional soil profile data. SoilGrids is a valuable resource for researchers, policymakers, and practitioners involved in soil-related studies and applications worldwide.

**Figure 1:**
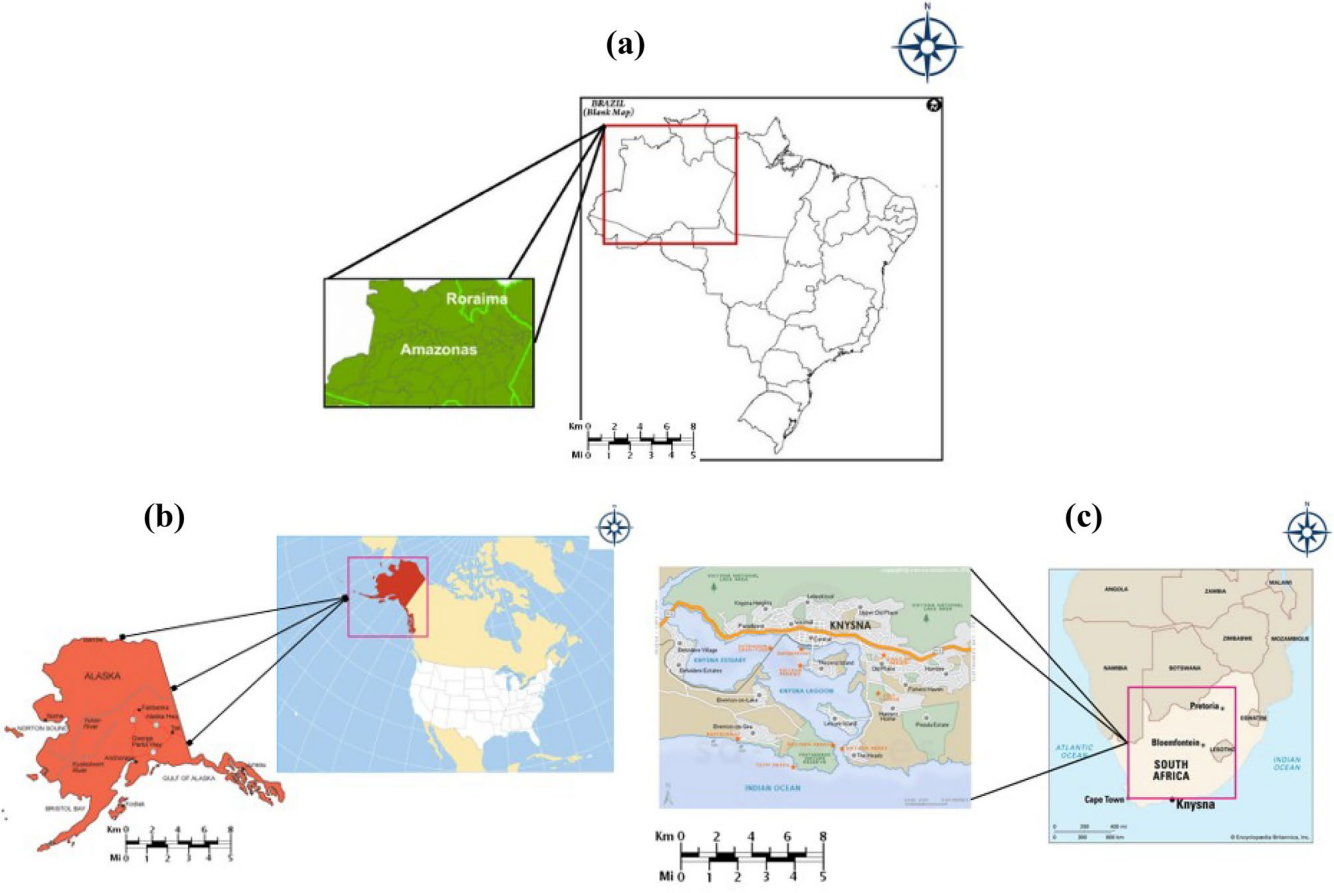
Study area: (**a**) Continental context—Brazil biogeographical zone Amazon, (**b**) Continental context—USA biogeographical zone Alaska, (**c**) Continental context—South Africa biogeographical zone Knysna. The map was generated with the QGIS v.3.28 software (https://qgis.org/en/site/).

The dataset constructed for vegetation recovery was downloaded from the USGS website mentioned above. It consisted of 1600 post-fire images from three central regions that were more suitable for our study namely: Amazon rainforest of Brazil with location co-ordinates 3.4653° S, 62.2159° W, Knysna region of South Africa with location co-ordinates 34.0351° S, 23.0465° E and Alaska with location co-ordinates 63.5888° N, 154.4931° W. Table 1 shows the collection of dataset used for processing our methodology. The construction of the dataset involved downloading Suomi NPP satellite’s EROS Visible Infrared Imaging Radiometer Suite (eVIIRS) data and then loading this in QGIS software, followed by creating an output layer that contains the NDVI values obtained by using the raster calculator present in the software. This layer is then exported to .jpeg format for model training and evaluation. This pre-processing step is performed for all the collected data and the obtained .jpeg file is added to the dataset. Figure 1 represents the location of our study area in a visualized map.

**Table 1:** Description of proposed method dataset.

Dataset size	Source of data	Location (s)	Type of data
3600	USGS	Amazon	eMODIS-NDVI (Image)
2000	Soil grid database	Worldwide	Soil information (CSV)
1600	USGS	Amazon, Knysna, Alaska	eVIIRS-NDVI (Image)

## Methodology

Wildfires cause lots of damage to ecology and economy, vegetation in particular is affected by sudden increases in temperature which can destroy trees, shrubs and herbs. Change in vegetation composition is possible as fire-adapted species tend to dominate over others that are less tolerant to fire, there is also a huge question mark over the vegetation regrowth possibilities in regions particularly affected by massive fires, and analysing recovery patterns after the fire is critical to understand the effects of fire and what all steps can be taken to improve the recovery if at all the recovery is less. So for this, we proposed an integrated framework involving Vegetation change detection by making use of sparse Autoencoders and a Deep Embedded Clustering (DEC) model, using a change map showing the difference between pre and post-fire images obtained, which is then embedded with the pre-fire image to show the change’s caused by fire; Vegetation Regrowth Assessment involves usage of Enhanced Vegetation Index (EVI) to get a time series representation of vegetation over the years through browning and greening fraction. Additionally, the regrowth possibility for a region is predicted by using soil data, which is fed as input to an Ensemble learning-based model. Finally, Vegetation Recovery Mapping is performed using AdaptiGAN.This unsupervised deep learning model, takes a pre-processed eVIIRS NDVI image as input and then provides the corresponding recovery map for that image.

**Figure 2:**
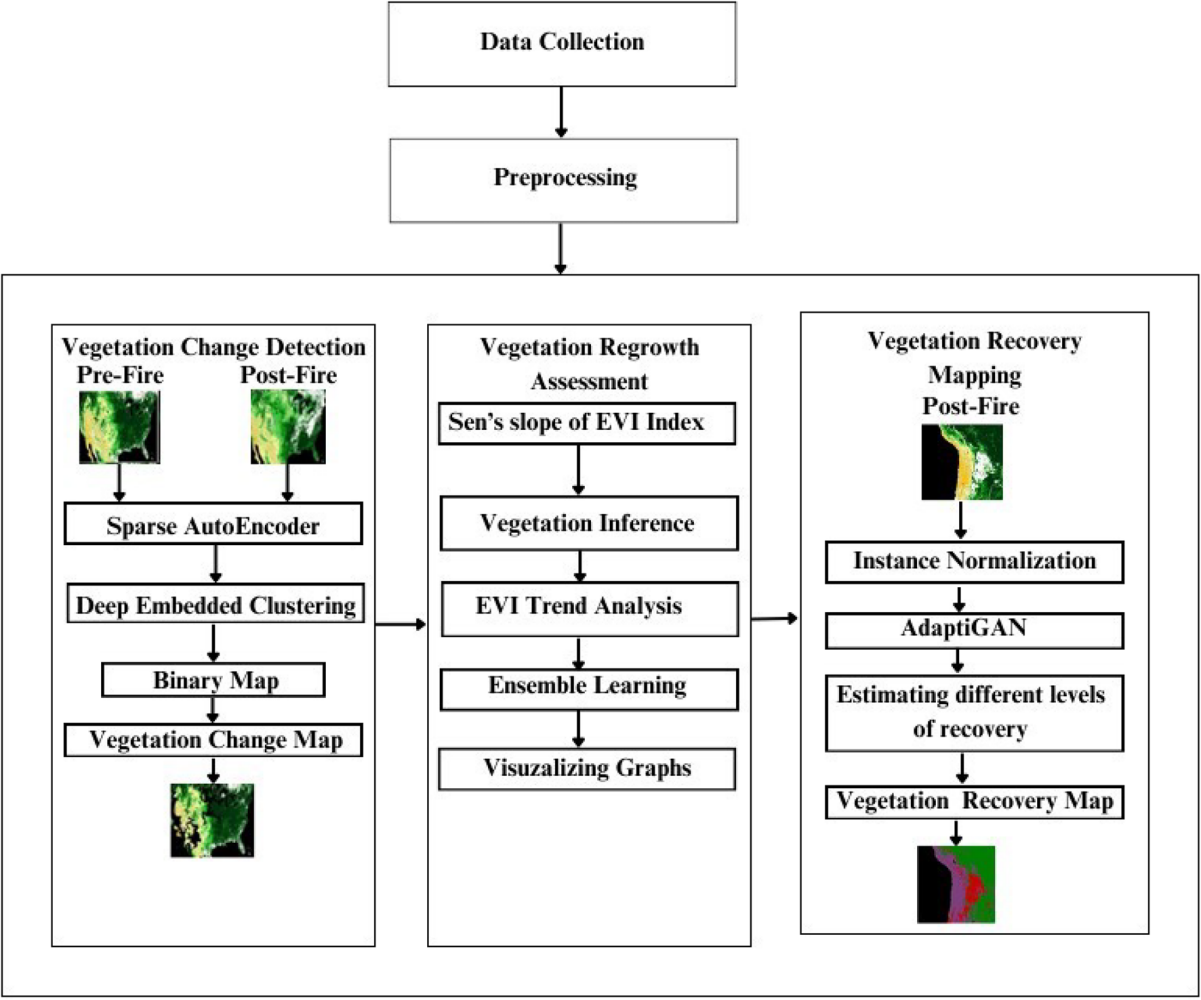
The Architecture Diagram for the proposed Approach. The map was generated with the QGIS v.3.28 software (https://qgis.org/en/site/).

The paper’s novelty lies in the comprehensive framework for assessing the effects of post-fire vegetation. The framework involves three significant modules: vegetation change detection using Sparse Autoencoders along with DEC, which provided an efficient alternative to pre-existing methodologies, followed by vegetation regrowth assessment using Ensemble learning and Sen’s slope followed by vegetation recovery mapping using a novel architecture named AdaptiGAN which is based on a deep learning algorithm with self-attention mechanisms and normalization techniques in place adding to its ability to generate accurate recovery maps. The architecture diagram for the proposed approach can be seen in Fig. 2.

### Change detection using deep embedded clustering (DEC)

A comprehensive assessment of long-term trends in vegetation change at the field scale is required for resource management and ecological assessment. Remote sensing data have been widely employed as the most remarkable change detection asset. The common approaches deployed for change detection include post-classification comparison, principal component analysis (PCA), and image differencing. New methods are required to use the more complex and diversified remote sensed data effectively that is anticipated to become so shortly via satellite and airborne sensors, which is still an active area of research.

**Figure 3:**
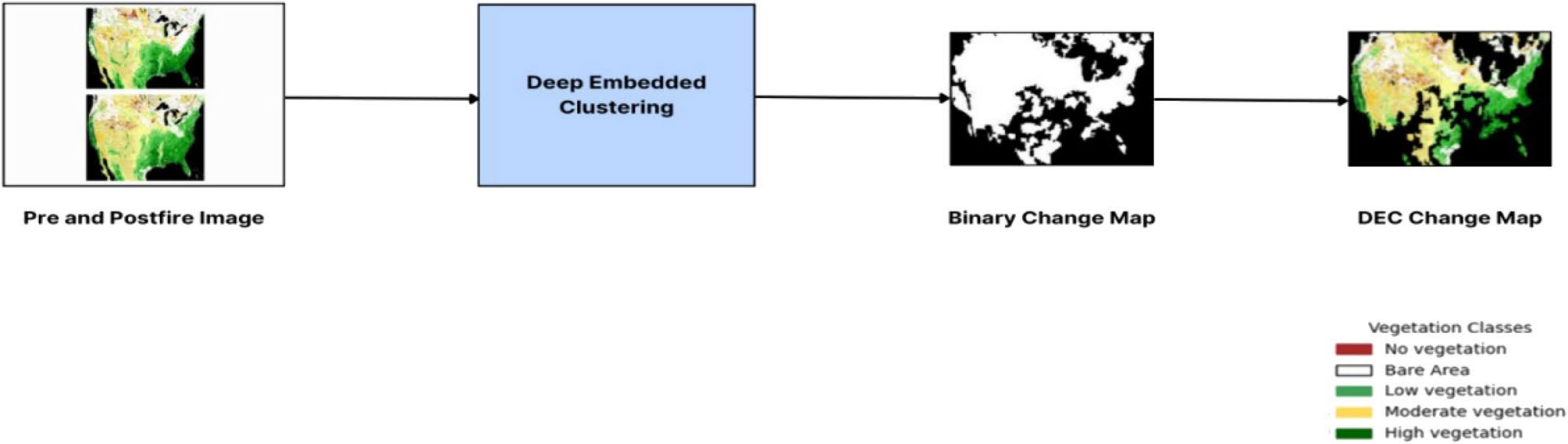
Framework of Vegetation Change Detection. The map was generated with the QGIS v.3.28 software (https://qgis.org/en/site/).

Vegetation change detection analyses the difference between satellite images obtained before and after the fire. The dataset comprises 3600 pre & post-fire images obtained from eMODIS NDVI v6. The QGIS Software is used calculates the NDVI index and splits the region into several classes of vegetation based on the index value of each pixel of the multi spectral image. The change detection process uses a Sparse Autoencoder to extract required features. Then, this representation is fed as input to the DEC model, an unsupervised learning technique used to iteratively group features, and ensuing assignments are used as supervision to update network weights. The model acquires feature representations through successive iterations by using labelled and unlabelled data points and alternatively finding target distributions from prediction. The distinction between images obtained before and after the fire in the forest region in Jefferson, California, is used to create the change map. Figure 3 represents the workflow of vegetation change detection.

### Splitting the classes

**Figure 4:**
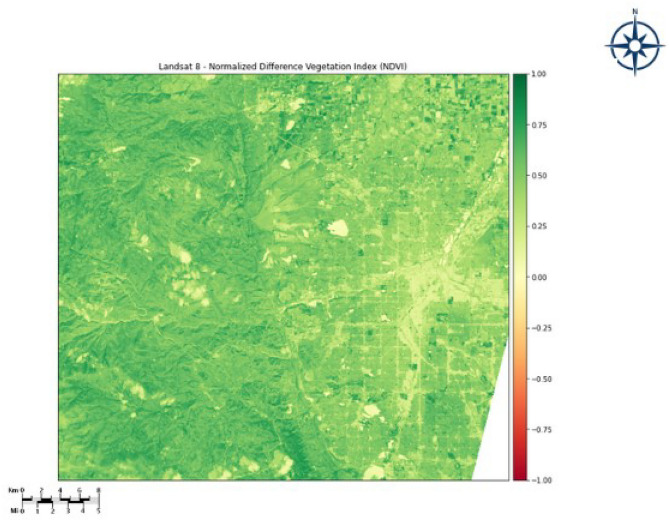
NDVI index class range ranging “− 1 to + 1”. The map was generated with the QGIS v.3.28 software (https://qgis.org/en/site/) and RGB satellite composite from Google Maps, layers available in QGIS.

**Figure 5:**
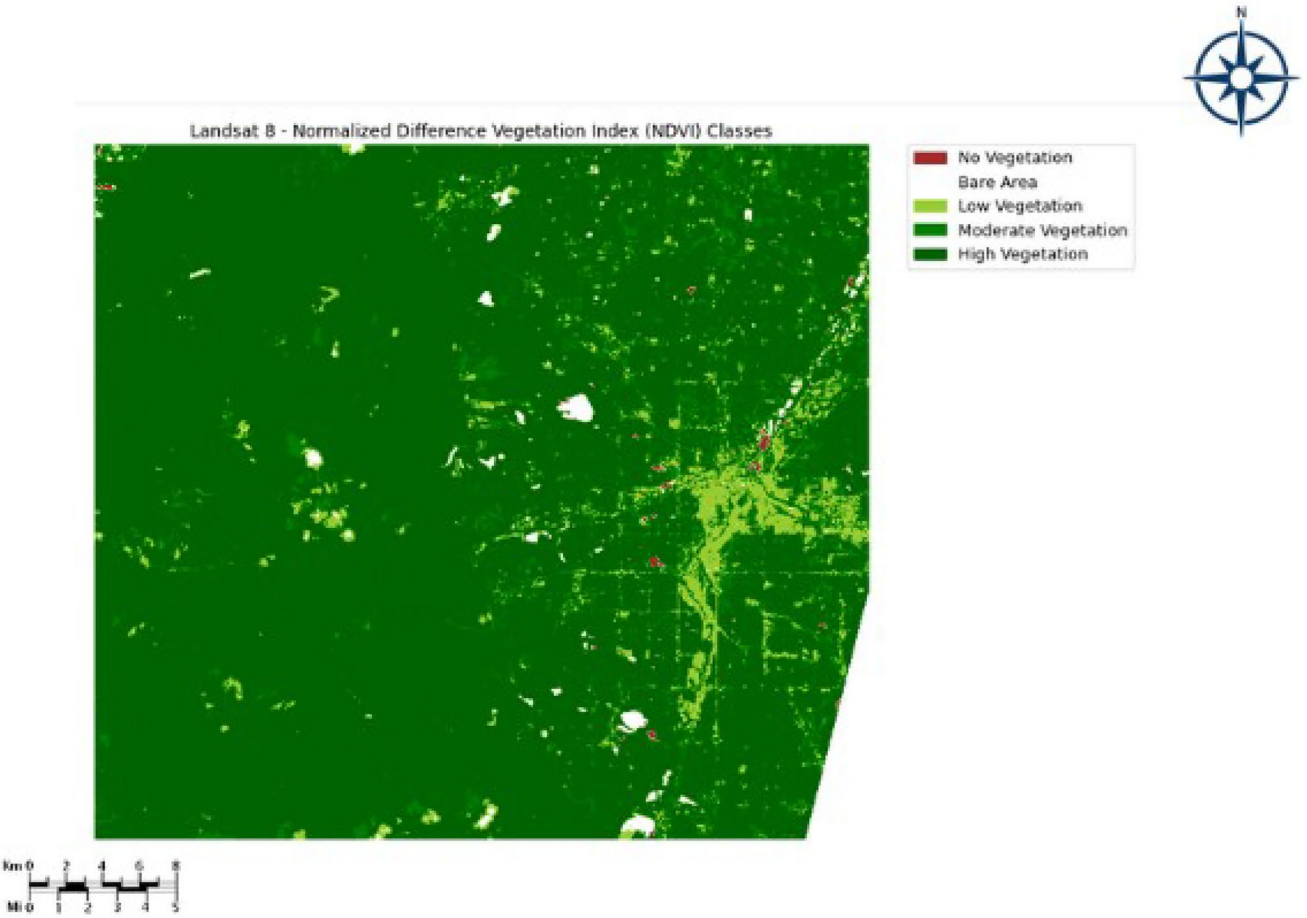
Classes generated from the NDVI index. The map was generated with the QGIS v.3.28 software (https://qgis.org/en/site/) and RGB satellite composite from Google Maps, layers available in QGIS.

The QGIS Software has been used to calculate the NDVI index and produce five different classes ranging from low vegetation to very high vegetation. Figure 4 below represents the NDVI index value ranging from “− 1 to + 1”. The Fig. 5 represents the set of classes generated from NDVI. Table 2 shows the Vegetation type and its associated NDVI Index. The threshold selection criteria are based on the NDVI values, which can range from − 1 to 1; now using this information we calculated the NDVI index and performed splitting of the region into several classes of vegetation based on the index value of each pixel of the multi spectral image. This processed image with the NDVI index served as the input for our DEC model.

**Table 2:** Vegetation and their corresponding NDVI ranges.

Vegetation	NDVI Index
No vegetation area	− 1 to 0
Bare area	0 to 0.1
Low vegetation area	0.1 to 0.25
Moderate vegetation area	0.24 to 0.4
High vegetation area	0.4 to 1

### Sparse autoencoder

An autoencoder represents a particular kind of Artificial Neural Network (ANN) typically used for unsupervised learning. An autoencoder that attains bottleneck information with an added restriction on sparsity is called a sparse autoencoder. The loss function is designed to push activations inside a layer. L1 regularization or Kullback–Leibler (KL) divergence between an appropriate distribution and anticipated mean neuron activation, such that sparsity constraint can be applied. In other words, the autoencoder minimizes the difference between the input and the reconstructed output and encourages sparsity in the activations of the hidden layer.

#### L1 regularization

By scaling the absolute value of the activation vector in layer h for observation i by a tuning parameter λ with its features x and x^, we may add a term to our loss function that penalizes it. The formula used can be seen in Eq. (1).

1 $$L\left(x,\widehat{x}\right)+ \lambda \cdot \sum_{i=1}^{n}i\cdot \Vert a\left(h\right)\cdot i\Vert$$

#### KL-divergence

KL-divergence is a measurement of variation in distribution over probability between two samples. A sparse parameter ρ represents the average activity of a neuron across a group of samples $\widehat{\rho }$. Neurons are induced to fire for a subset of observations by limiting the average activity of a neuron and differentiating j it over a group of samples. To contrast the expected distribution to actual distributions over all the hidden layer nodes, we can define it as a Bernoulli random variable distribution and use the KL divergence. The formula used can be seen in Eq. (2).

2 $$L\left(x,\widehat{x}\right)+ \sum_{j=1}^{n}jKL(\rho ||\widehat{\rho }j)$$

### Deep embedded clustering

**Figure 6:**
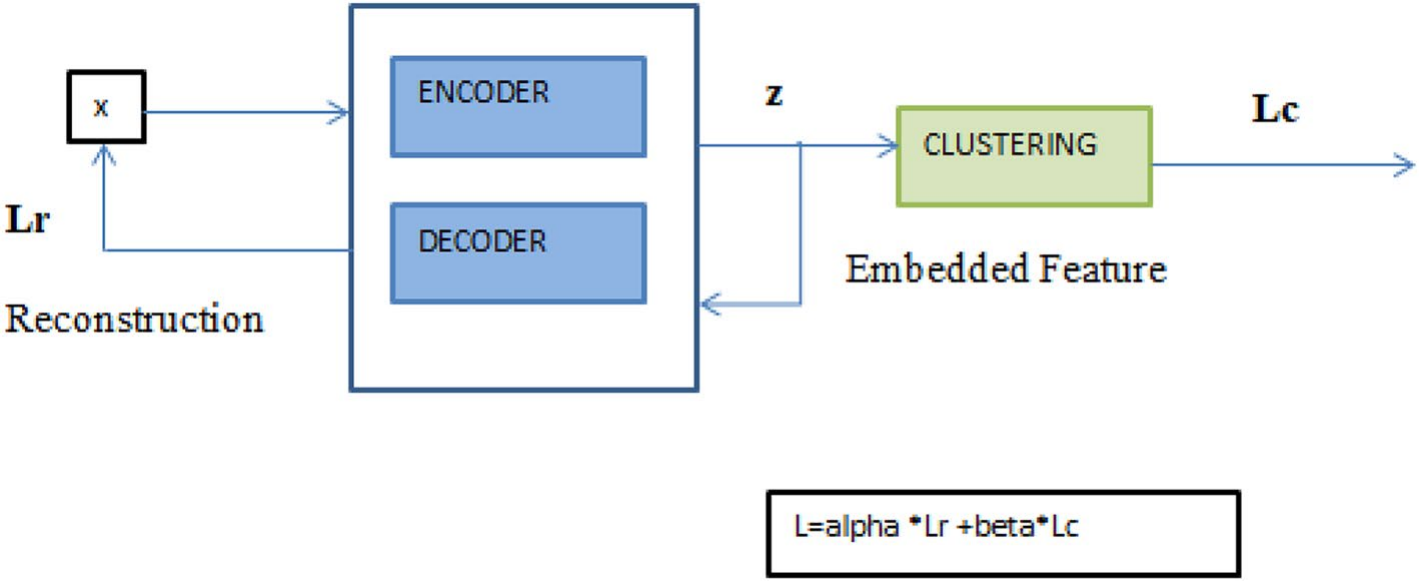
Block diagram of DEC.

The model DEC works in two phases:

- Initialize phase with deep sparse autoencoders
- Clustering, where they successively repeat calculating a supplementary target distribution and minimize the KL divergence associated. Figure 6 represents the block diagram of Deep Embedded Clustering. It briefs the model’s workflow, which comprises an Autoencoder and Clustering block. A soft assignment is initially calculated between cluster centroids (vegetation classes) and embedded points. Then, t-distribution computes the similarity index between embedded points and centroid t-distribution is used as a non-parametric representation to identify the similarity index between embedded points. One can upgrade deep mapping and improve the cluster’s centroids by employing an auxiliary target distribution and learning from recent high-confidence assignments. In particular, soft assignment is matched to target distribution to train the model and centroid of clusters.

This change detection process’s basic workflow is that images obtained before and after a fire are given as inputs simultaneously to our DEC model. A Sparse autoencoder is used to extract features and pass them to the clustering function where each of its similar features is aggregated into a cluster. It gives a binary map of pre & post-fire images, by finding the difference between the images obtained before and after the fire we get our change map as the final output, which in return will be embedded with a pre-fire image to produce changes in the vegetation of the region. DEC model output is not NDVI, but rather the input is. The input image is constructed based on the 5 NDVI classes (No, Bare, Low, Moderate and High), which are then fed as input to obtain the binary change map and DEC change map as output.

The DEC (Deep Embedded Clustering) model generates binary burned maps due to its integration of deep sparse autoencoders alongside clustering techniques. Autoencoders play a crucial role in DEC by providing a mechanism for unsupervised feature learning and dimensionality reduction. Specifically, the deep sparse autoencoders in DEC are designed to learn meaningful representations of the input data while enforcing sparsity in the activations of the hidden layers. This capability is essential for capturing intricate patterns and features in satellite imagery, including those indicative of burned areas. By leveraging autoencoders, DEC can compress the input data into a lower-dimensional latent space, facilitating more efficient clustering and representation learning. Additionally, combining autoencoders with sparsity constraints, KL divergence, and L1 regularization further enhances DEC's ability to extract relevant features and enforce sparsity within the hidden layers, thereby aiding in accurately identifying burned areas in satellite imagery. Overall, integrating autoencoders within the DEC framework provides a comprehensive and effective approach for generating binary burned maps from satellite imagery, leveraging the strengths of both deep learning and clustering techniques.

**Figure:**
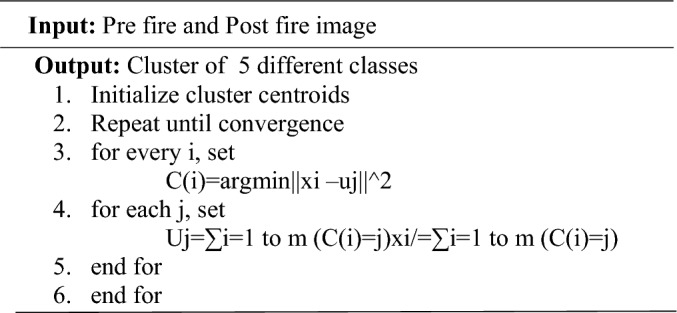
**Algorithm.**

### Ensemble learning

Vegetation regrowth assessment following a wildfire is essential to post-fire monitoring and ecosystem recovery. Sen’s slope, also termed Sen’s estimator or Sen’s method, is a statistical technique used to assess trends or changes in time series data. While Sen’s slope is typically used for analysing trends in various fields, such as hydrology and climate science, it can also be applied to assess vegetation regrowth following wildfires. This involves obtaining time series data representing vegetation indices or other relevant vegetation metrics for the study area. This data should span multiple periods, before and after the wildfire event.

**Figure 7:**
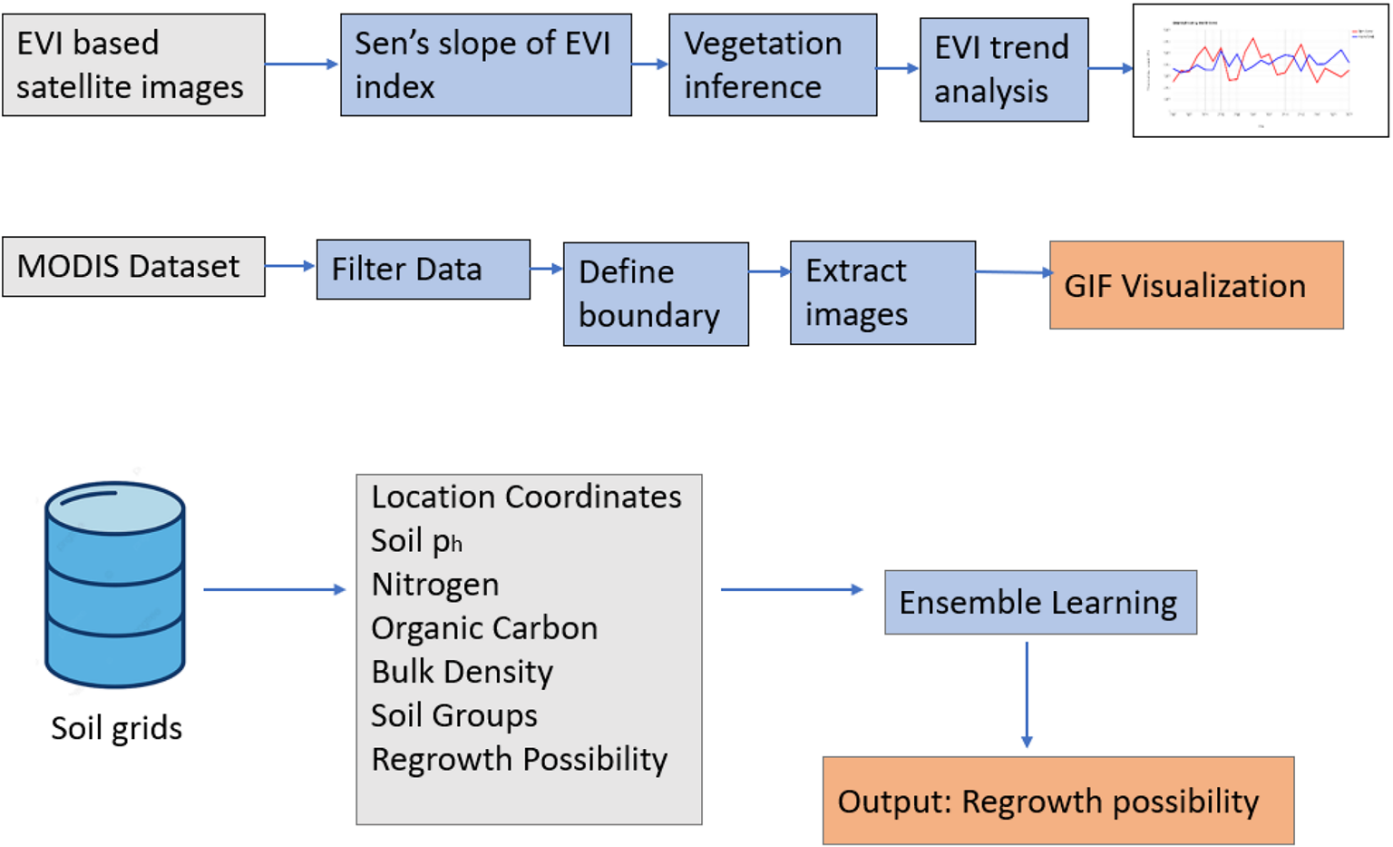
Workflow of vegetation regrowth assessment.

Sens’s slope of the EVI index is calculated. Based on the magnitude of the slope, browning and greening fraction can be determined. The trend of the EVI pattern over the years is analysed and it is visualized as a graph. MODIS dataset has been acquired for different regions and their NDVI images are converted to RGB frames and aggregated to provide animation representing changes in vegetation over the years. The prediction of regrowth possibility involves collecting soil data from the soil grid database, which is then trained using Ensemble learning to provide an outcome. Figure 7 represents the workflow of vegetation regrowth assessment.

The working of Vegetation Regrowth Assessment using Sen’s slope can be described in the following steps.

- **Data collection**: Remote sensing data such as satellite images from the MODIS dataset is collected for a specific area over a period of time.
- **Pre-processing**: Pre-processing the remote sensing data eliminates noise or artifacts and converts it into a usable format. This may involve cloud removal, atmospheric correction and radiometric calibration.
- **Vegetation Index Calculation**: The vegetation index, such as the Enhanced Vegetation Index (EVI), is calculated from pre-processed remote sensing data.
- **Time-series analysis**: Vegetation index values for each time step are analysed using Sen’s slope estimator to calculate the trend or slope of the vegetation index over time. The result approximates the pace at which an area’s vegetation is growing again or decreasing.
- **Visualization**: The analysis results can be visualized using various techniques, such as a time series plot of the vegetation index or a map showing the spatial distribution of the vegetation regrowth or decline.

Vegetation Regrowth Assessment using Sen’s slope is an excellent technique for providing insights into the natural regeneration steps of vegetation. It also allows the identification of areas that may need interventions to support recovery in a region, after a disaster like wildfire.

### Plotting trends based on EVI

The following steps describe the process of Plotting trends based on EVI values calculated over a period of time:

- The World Database on Protected Areas (WDPA) dataset is the foundation for creating a MODIS 250 m/pixel 16-day composite vegetation indices dataset.
- Add images for every year between 2000 and 2002 to create a collection.
- Every one of the images has been computed to attain the highest EVI throughout every month of respective years.
- This is an annual examination of the state of vegetation. Add the year as a band to prepare for a through linear-trend analysis.
- Determine each pixel’s sen’s slope of highest summer EVI over time to estimate a linear trend.
- Compute and display the regression slope values as histograms.
- We can determine the browning and greening fraction of the vegetation by measuring the slope’s value.

Sen’s slope = Median {$\frac{{x}_{j}-{x}_{i} }{j-i}$: i < j}.

**Figure 8:**
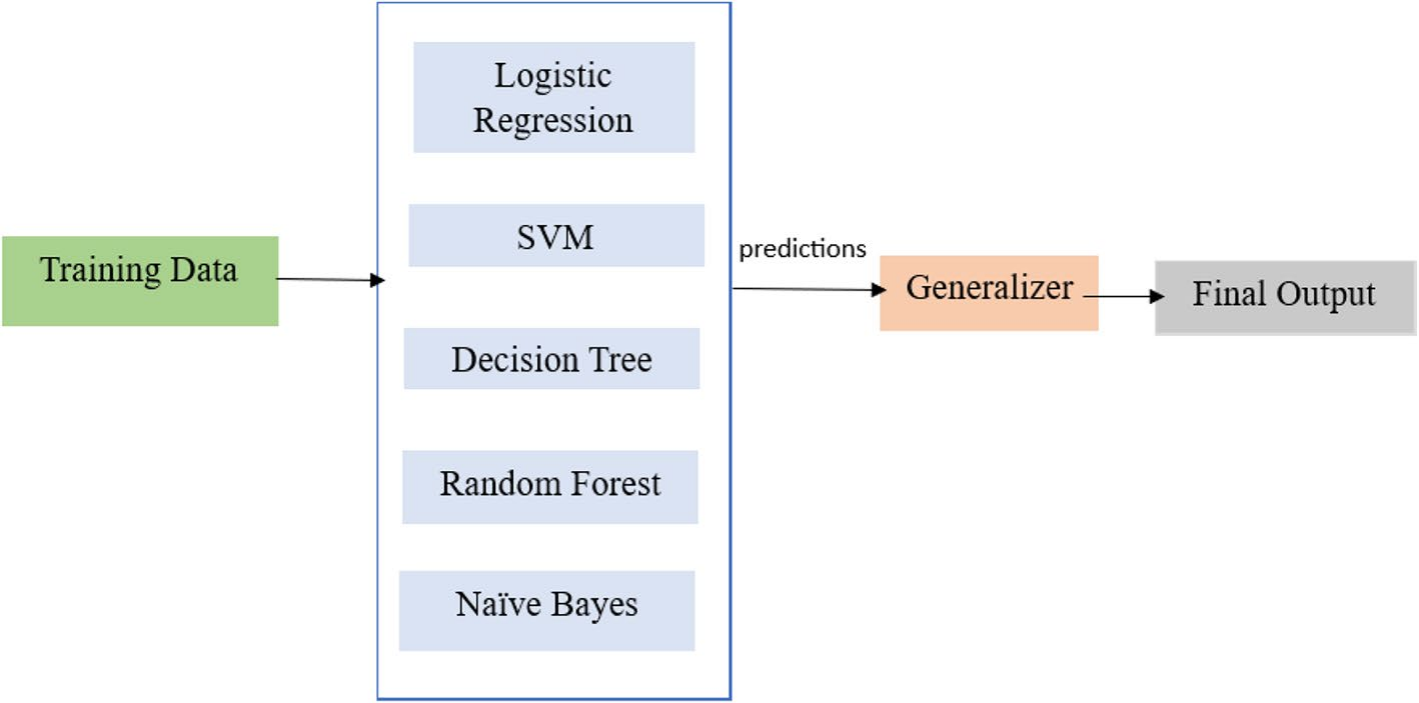
The proposed ensemble learning approach.

The dataset for soil properties has been acquired from the soil grid database. Stacking is an Ensemble machine learning algorithm that combines results from multiple models. In the proposed work, we consider simple machine learning algorithms, including Logistic Regression, SVM, Decision Tree, Random Forest and Naïve Bayes, as weak models and their predictions are given to the generalizer to provide the outcome of regrowth possibility. Figure 8 below shows the Ensemble learning approach using stacking. So, the basic architecture of the utilized Stacking process is:

- **Base models (Level 0)**: This involves training diverse base models on the training data, in this case Logistic Regression, SVM, Decision Tree, Random Forest and Naïve Bayes.
- **Meta-model (Level 1)**: In our case, a meta-model the Generalizer, is trained on the predictions from the base models. This model learns to combine the base model’s predictions to generate a final prediction.
- **Final prediction (output)**: Based on the soil properties as input, the trained model makes predictions about the possibility of regrowth in a region.

### Adaptive generative adversarial network (AdaptiGAN)

Vegetation Recovery Mapping provides valuable information about the ecological, environmental, and management aspects of post-fire landscapes. Traditional approaches involving field surveys and ground truthing can be cumbersome and expensive while simultaneously providing a need for a more efficient strategy to speed up this process. This involves using remote-sensing technologies such as satellite imagery combined with spectral indices like NDVI and deep learning techniques. Now, using both remote sensing technologies and deep learning together can provide efficient and accurate results that can be used for future planning like resource allocation and habitat restoration in fire affected regions.

**Figure 9:**
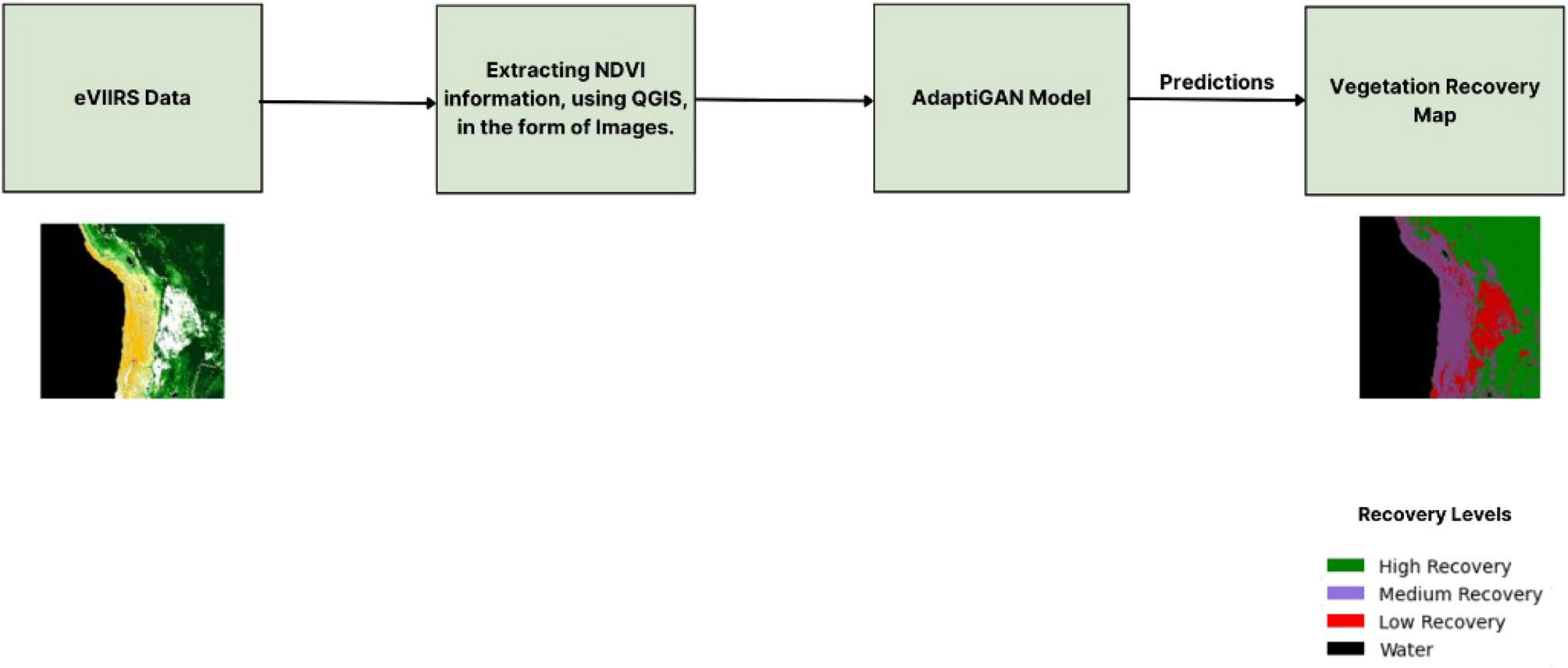
Framework of vegetation recovery mapping. The map was generated with the QGIS v.3.28 software (https://qgis.org/en/site/).

For this purpose, the proposed approach collects satellite data from e-VIIRS products obtained from Suomi NPP satellites, of different fire affected regions. These are then pre-processed using the QGIS tool, through which NDVI is calculated and an image like representation is obtained. This dataset serves as the training data for an unsupervised learning algorithm called AdaptiGAN, which can be adapted based on the data we feed into it. Using this trained model, we can obtain recovery maps for regions affected by fire for various time periods. Figure 9 represents the framework for the Vegetation Recovery Mapping Approach.

**Figure 10:**
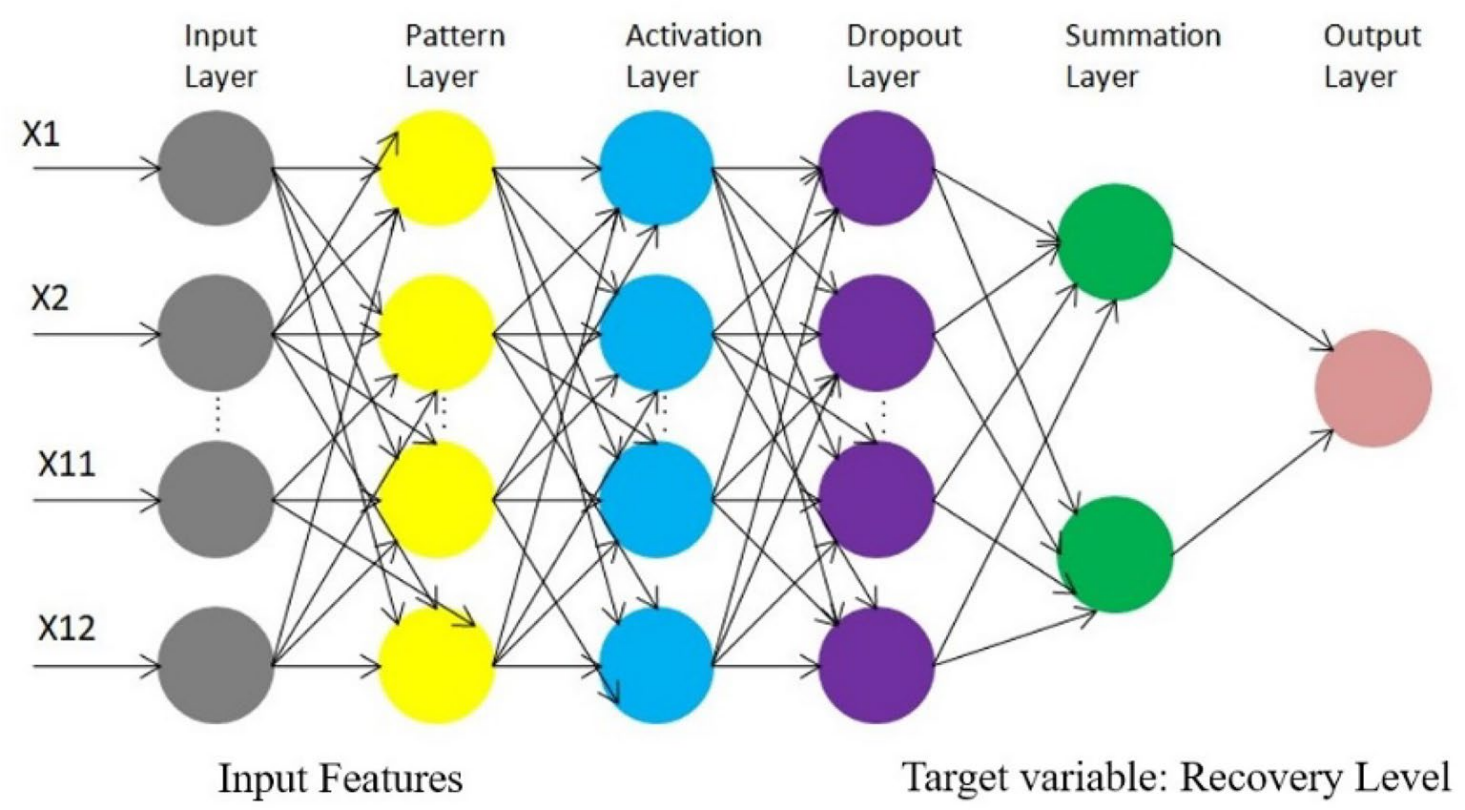
Network architecture of AdaptiGAN.

The AdaptiGAN is neural network architecture, a Generative Adversarial Network (GAN), whose generator follows an Encoder-Decoder architecture making use of self-attention mechanisms for recording long-range dependencies which in turn will help in improving feature representation and the discriminator makes use of the PatchGAN architecture which helps in capturing fine-grained details of the input images. Self-attention mechanisms and normalization techniques are instrumental in capturing domain-specific features, which is very important in our case of generating recovery maps. The critical components related to this architecture can be seen in more detail below, and the network architecture can be seen in Fig. 10.

### Components in the AdaptiGAN architecture

#### Generator

This follows encoder-decoder architecture. The encoder downsamples the input image to extract features, and the decoder upsamples these features to get the final output image. The downsample layers reduce spatial dimensions and increase the number of channels capturing hierarchical features, while the upsample layers increase spatial dimensions, enabling the generation of a high-resolution image. A self-attention mechanism is introduced after the third downsampling layer to capture long-range dependencies and improve feature representations.

#### Discriminator

The discriminator employs PatchGAN architecture, classifying local patches of the input images as real or fake. This helps in capturing fine-grained details. Convolutional layers with leaky ReLU activations are used for feature extraction and discrimination. Batch normalization is applied to normalize the activations, aiding in the stability and training of the discriminator.

#### Weight regularization

L2 weight regularization is applied to the generator and discriminator’s convolutional layers. This helps to fend off overfitting and improves the architecture’s generalization.

#### Instance normalization

Instance normalization is applied in the discriminator after the second and third convolutional layers. It normalizes activations across channels and spatial dimensions independently for each sample.

#### Dropout

Dropout is applied in the generator after the concatenation of feature maps during decoding process. It helps regularize the network by randomly dropping a fraction of the units during training.

#### Activation functions

ReLU activation is used in various parts of both the generator and discriminator to introduce non-linearity, while Leaky ReLU is used in the discriminator to allow a small, non-zero gradient when the input is negative.

#### Summation

The generator uses summation layers to combine feature maps from different stages, aiding in the generation of detailed and realistic images.

#### Output layer

The generator has a tanh-activated convolutional layer in the output to produce the final generated image.

**Figure:**
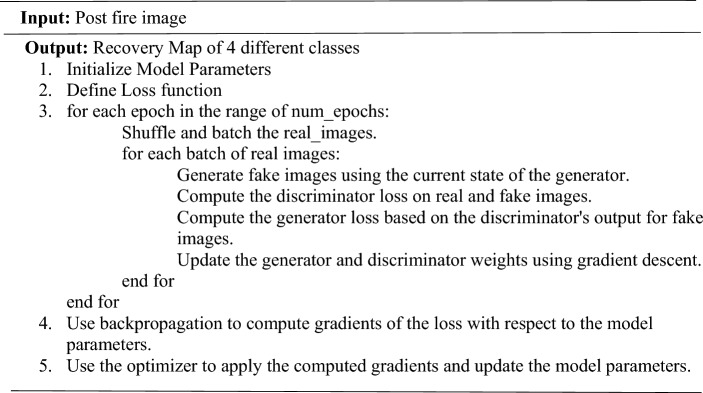
**Algorithm.**

## Results and discussion

To verify the viability of our proposed approach, the performance of the three modules was tested. There are two areas for the experimental equipment: one for testing and the other for training. In the training phase, the deep learning model and machine learning model used for this research were trained in the Google Collaborator, which provided a hosted Jupyter notebook with a Python environment implemented on a server with AMD® 7000 Series Ryzen™ 9 7950X CPU @(5.7 GHz) with 16 GB memory, Radeon RX 7800 XT (GPU) with 16 GB of memory.

For testing, performance analysis was performed on the vegetation change detection module by using metrics like precision and recall. This was performed on the same Google Colab platform with a Python environment. The vegetation regrowth assessment module was then deployed using a streamlit web application to display the results for the possibility of vegetation regrowth in a region, which provided an interface where users can enter Location details and soil data to predict regrowth possibility. This can be validated by cross-referencing the exact location in the USGS Earth Explorer tool to check for vegetation,forthe vegetation recovery mapping module, metrics like Mean Squared Error (MSE) and Mean Absolute Error (MAE) were used. The server used for this testing environment is AMD® 7000 Series Ryzen™ 9 7950X CPU @(5.7 GHz) with 8 GB memory, Radeon RX 7800 XT (GPU) with 8 GB of memory.

### Experimental results and analysis for vegetation change detection using deep embedded clustering

**Figure 11:**
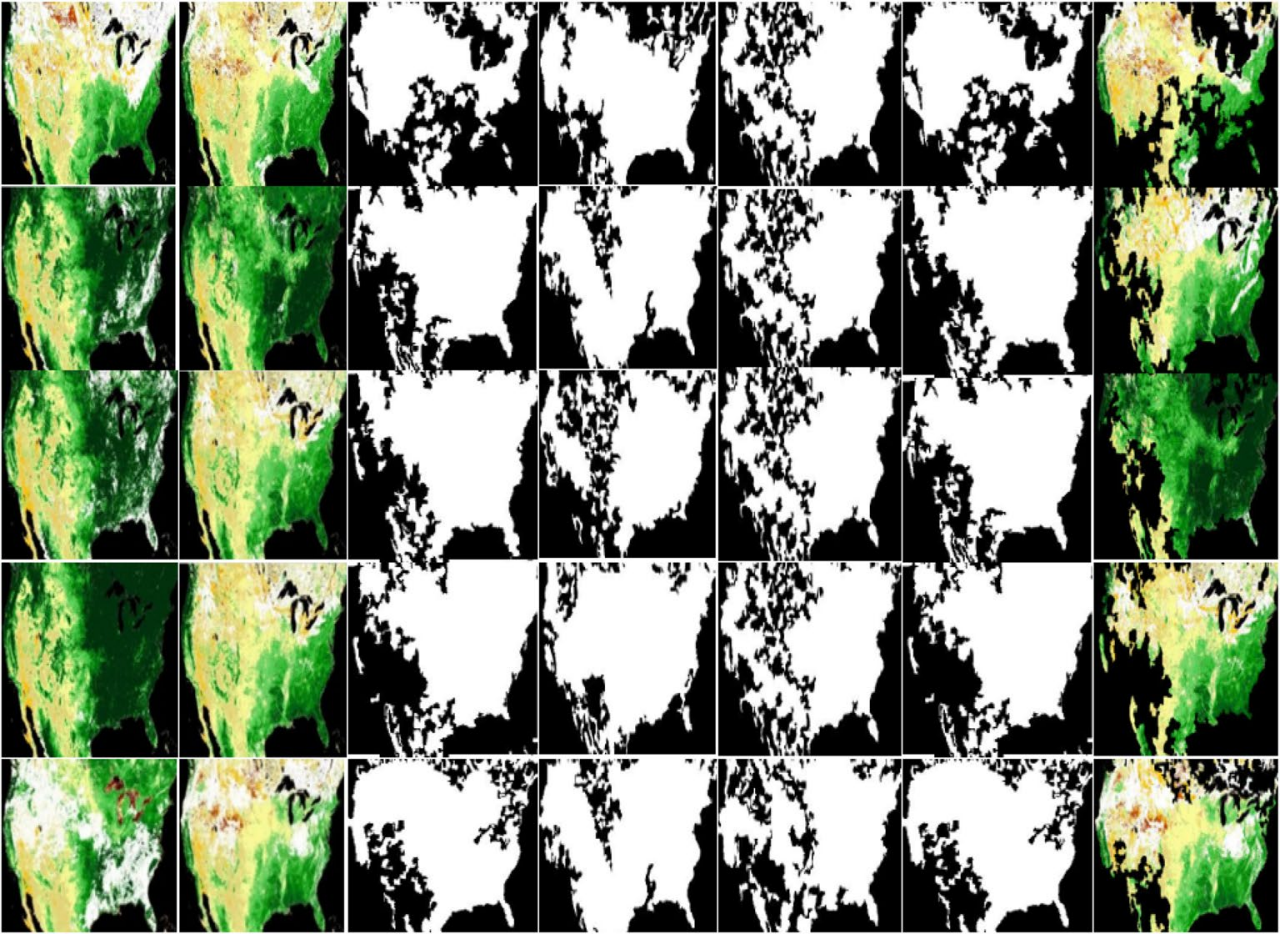
Visual comparison of various change detection models. From left to right: original pre-fire, original post-fire, Ground truth, STA Net, Bi-attention SFA, change map of DEC and DEC. The map was generated with the QGIS v.3.28 software (https://qgis.org/en/site/).

Vegetation change detection to assess long term trends in vegetation change, we propose the DEC model. Change detection is analysed by finding the difference between satellite-based imagery obtained before and after the fire. The dataset comprises 3600 pre-fire and post-fire images obtained from eMODIS NDVI v6. Figure 11 shows qualitative comparisons of DEC with other deep convolutional models.

Vegetation change detection performance is evaluated based on Precision, Recall, F1 and Accuracy (ACC), compared to other trained models. The DEC model achieved an impressive accuracy of 96.17%. Table 3 Comparison results between existing approaches such as STA Net, Bi-attention SFA, and our proposed model DEC. The deep embedded clustering is evaluated based on the loss metrics that contribute to minimizing the KL divergence. The proposed change detection model shows a loss of around 18.57, considerably lower than existing approaches. Table 4 Loss metrics were compared between existing models such as STA Net, Bi-attention SFA and the proposed DEC model.

**Table 3:** Comparison results of various deep convolutional network models for wildfire prediction.

Model	Precision (%)	Recall	F1 (%)	Accuracy (%)
STA Net	81.25	82.38	85.68	87.15
Bi-attention SFA	82.59	83.79 %	87.32	88.60
DEC	90.07	91.45 %	93.72	96.17

**Table 4:** Loss tabulation metrics for vegetation change detection.

Model	Loss
STA Net	26.27
Bi-attention SFA	26.77
DEC	18.57

The deep embedded clustering is compared with the various other change detection models and justified with minimal loss value after the successful completion of epochs.

### Experimental results and analysis for vegetation regrowth assessment using ensemble learning

**Figure 12:**
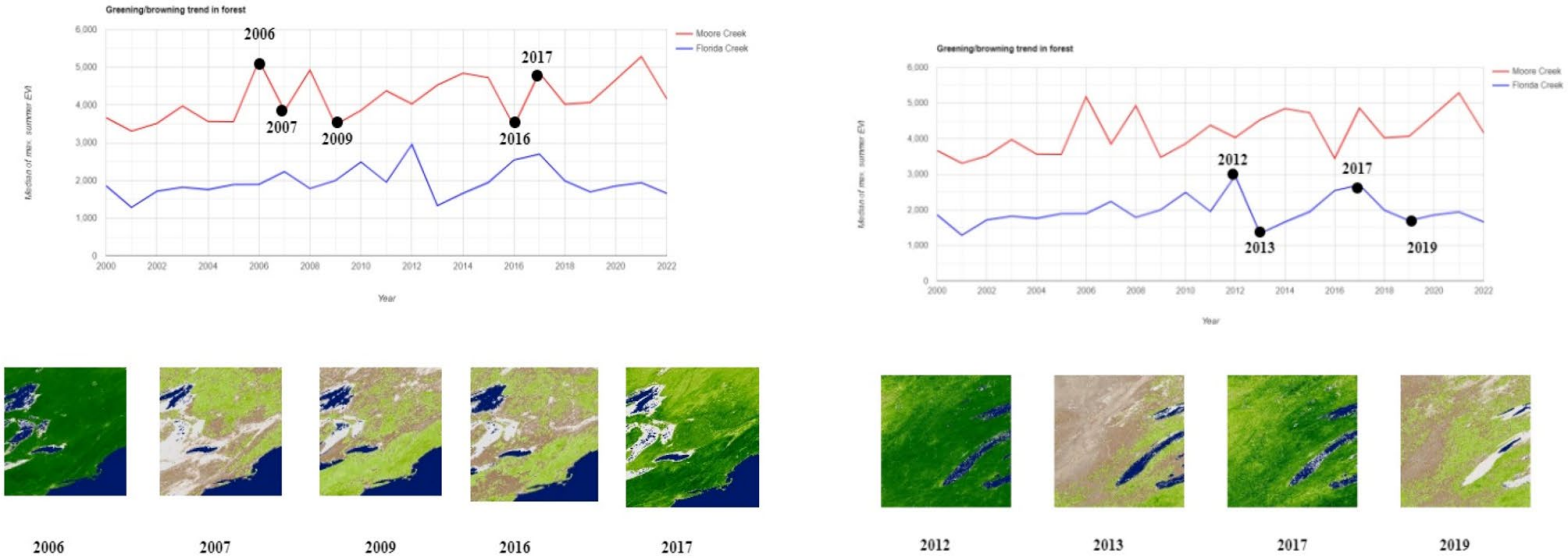
Browning and greening fraction of EVI trend for Moore Creek and Florida Creek. The map was generated with the Google Earth Engine v.7.3 software (https://earthengine.google.com/). Plots were generated using Python’s Matplotlib library, version 3.7.0 (https://matplotlib.org/3.7.0/).

**Figure 13:**
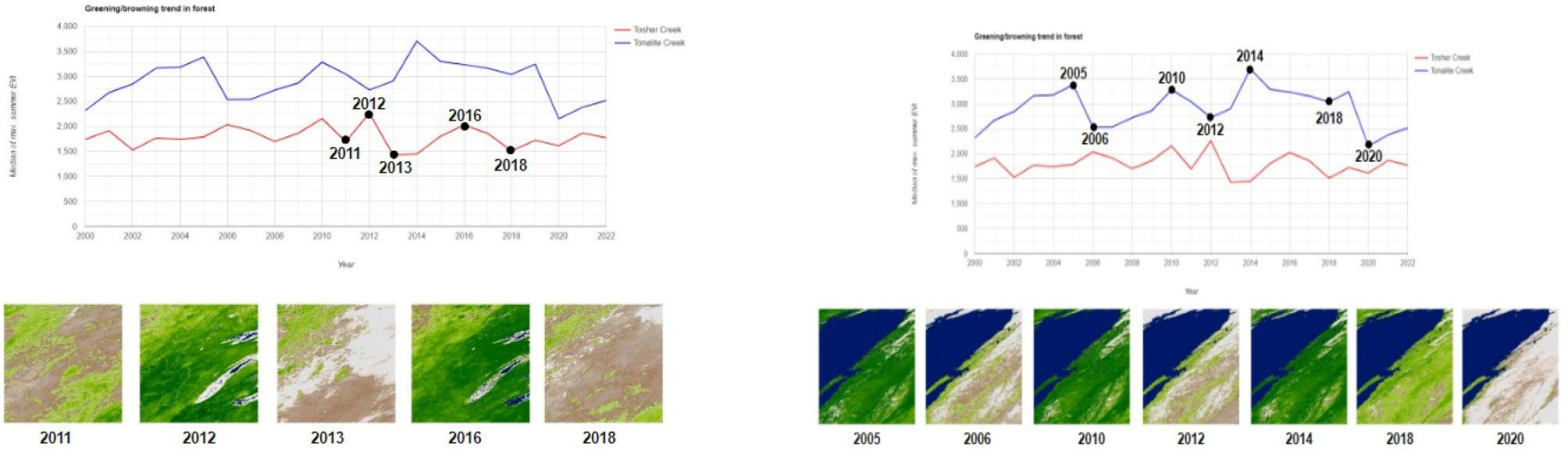
Browning and greening fraction of EVI trend for Tosher Creek and Tonalite Creek. The map was generated with the Google Earth Engine v.7.3 software (https://earthengine.google.com/). Plots were generated using Python’s Matplotlib library, version 3.7.0 (https://matplotlib.org/3.7.0/).

After a wildfire, recovery of an ecosystem and post-fire monitoring rely primarily on vegetation regeneration. Sen’s slope estimator is used to calculate the trend of the EVI pattern over the years and it is visualized as a graph. The browning and greening fraction is determined based on the magnitude of the slope. Figures 12 and 13 EVI trend graph of Moore Creek, Florida Creek, Tosher Creek and Tonalite Creek, along with its browning and greening fraction for the respective area over the years. The years differ because exact coverage and fluctuations should be visible, since we have chosen different regions, they will not be affected simultaneously. We collected data over a span of ten years for different creeks. As mentioned above, the graph-based visualization will not be accurate since all areas are different due to different fire timelines for different regions. So, to plot graphs we can conclude that changes are present over a period of time. Hence, to visualize all this, we mentioned years differently. Table 5 Tabulation comparison of browning and greening fraction with its sq. km for Moore, Florida, Tosher and Tonalite Creek. Additionally, the year’s corresponding to comparison results provided in the table differ for different creeks, in the case of Moore Creek it ranges from 2006 to 17, Florida Creek it ranges from 2012 to 19, Tosher Creek from 2011 to 18 and Tonalite Creek 2005–2020.

**Table 5:** Comparison results of browning and greening fraction using Sen’s slope.

Name	Years	Browning fraction	Browning sq. km	Greening fraction	Greening sq. km
Moore Creek	2006–2017	0.284	0.529	0.978	0.154
Florida Creek	2012–2019	0.106	0.362	0.8950	3.066
Tosher Creek	2011–2018	0.723	0.945	0.28	0.365
Tonalite Creek	2005–2020	0.45	18.197	0.553	22.368

**Figure 14:**
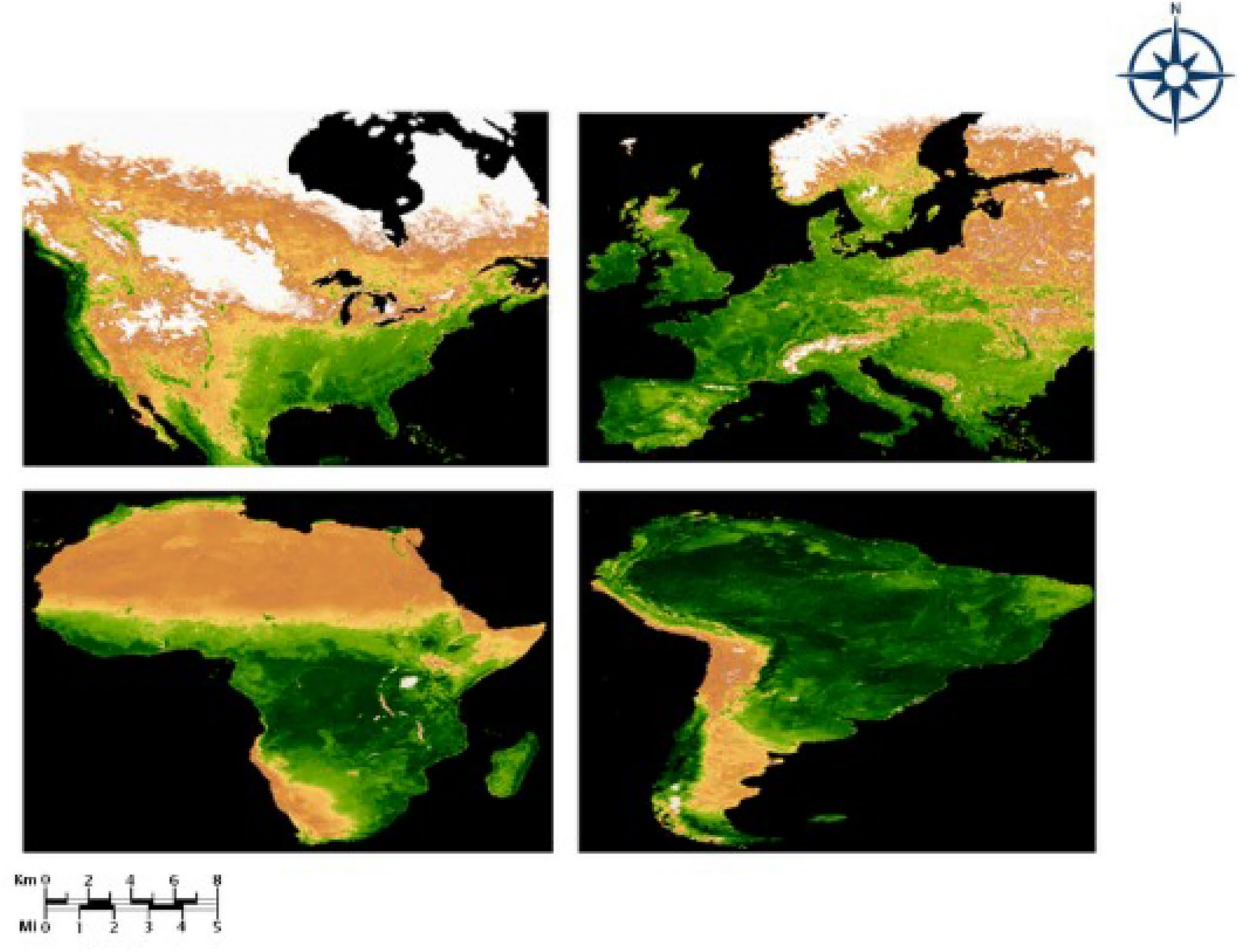
Results from QGIS application. NDVI images for different regions. The map was generated with the Google Earth Engine v.7.3 software (https://earthengine.google.com/).

The dataset for vegetation regrowth assessment is collected from Google Earth Engine fetched from the MODIS dataset. Sen’s slope is used to estimate the browning and greening fraction. It is used to analyse the trend of EVI patterns as well. The sections below show the tabulated results and graphs of various other models and proposed systems, thereby adding a justification. Ensemble learning can be used to provide the output for regrowth. Figure 14 shows the NDVI images obtained after processing them using the QGIS application.

**Figure 15:**
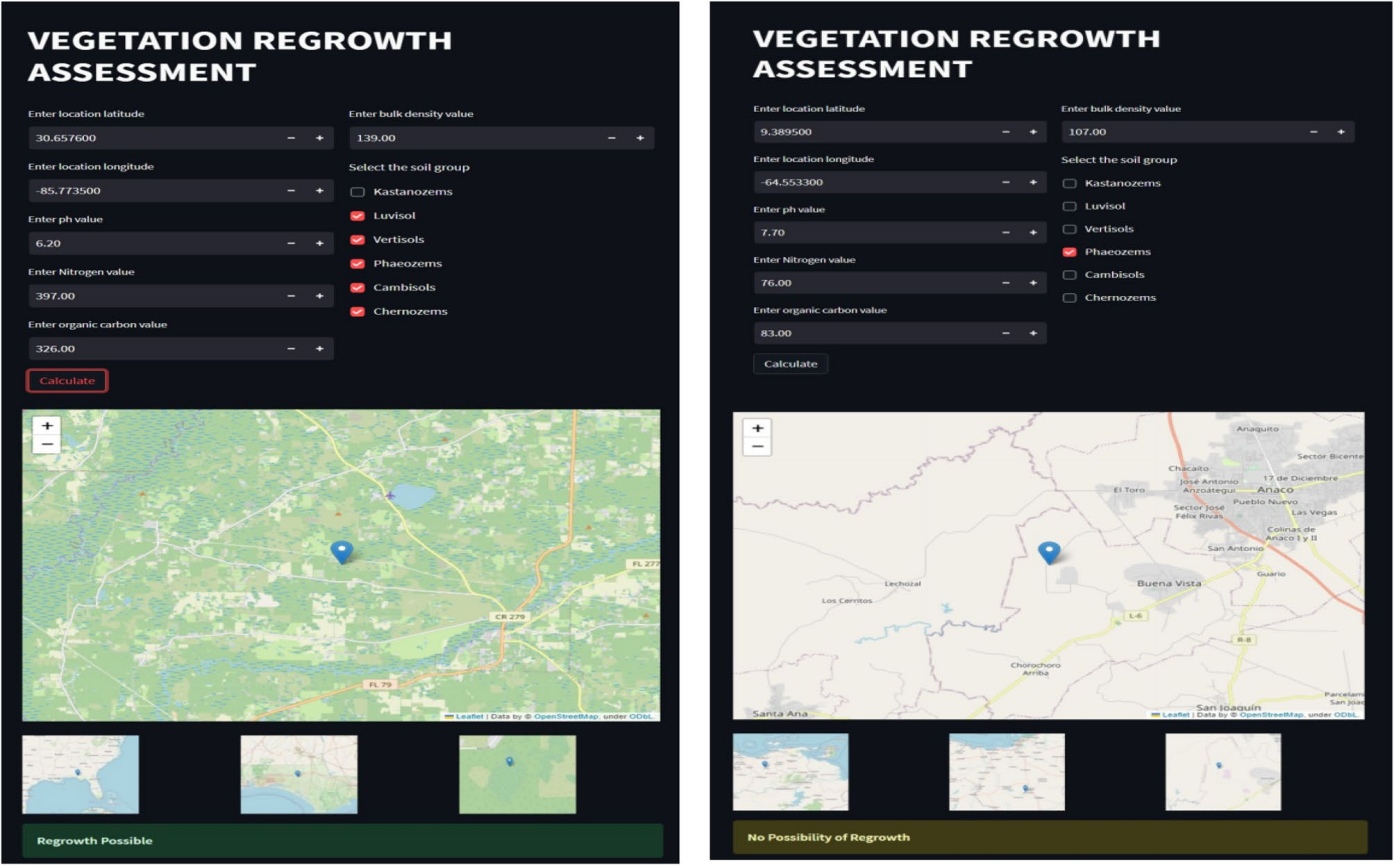
Results from streamlit application. Left to right: Possibility of regrowth and Not-possibility of regrowth. The map was generated with the Open Street Maps v0.7.60 API (https://www.openstreetmap.org/) and EarthExplorer (https://earthexplorer.usgs.gov/).

The possibility of regrowth is predicted by collecting and training soil properties using Ensemble learning to estimate its results. The performance of the system is tested using the Streamlit platform; it is deployed to display results for the possibility of vegetation regrowth. Users can enter the location coordinates (latitude and longitude), pH value, nitrogen value and soil group from where data are fetched and the possibility of prediction is estimated. Figure 15 shows the results obtained from the streamlit application, which predicts the possibility and not-possibility of vegetation regrowth. Table 6 represents the overall evaluation results of the vegetation regrowth assessment.

**Table 6:** Performance metrics for vegetation regrowth assessment.

References	Methodology	Precision (%)	Recall	F1 (%)	Accuracy (%)
Chen et al. (2021)^37^	Random forest	84.25	86.78	85.64	88.16
Singh et al. (2016)^38^	SVM	87.15	89.34 %	88.45	90.23
Ogungbuyi et al. (2023)^39^	Linear regression	89.27	88.54 %	89.12	91.56
Qiu et al. (2021)^40^	VCT-SVM	90.23	87.89 %	89.65	92.00
Proposed approach	Ensemble learning	94.03	92.45 %	95.43	97.19

### Experimental results and analysis for vegetation recovery mapping using adaptive generative adversarial network (AdaptiGAN)

**Figure 16:**
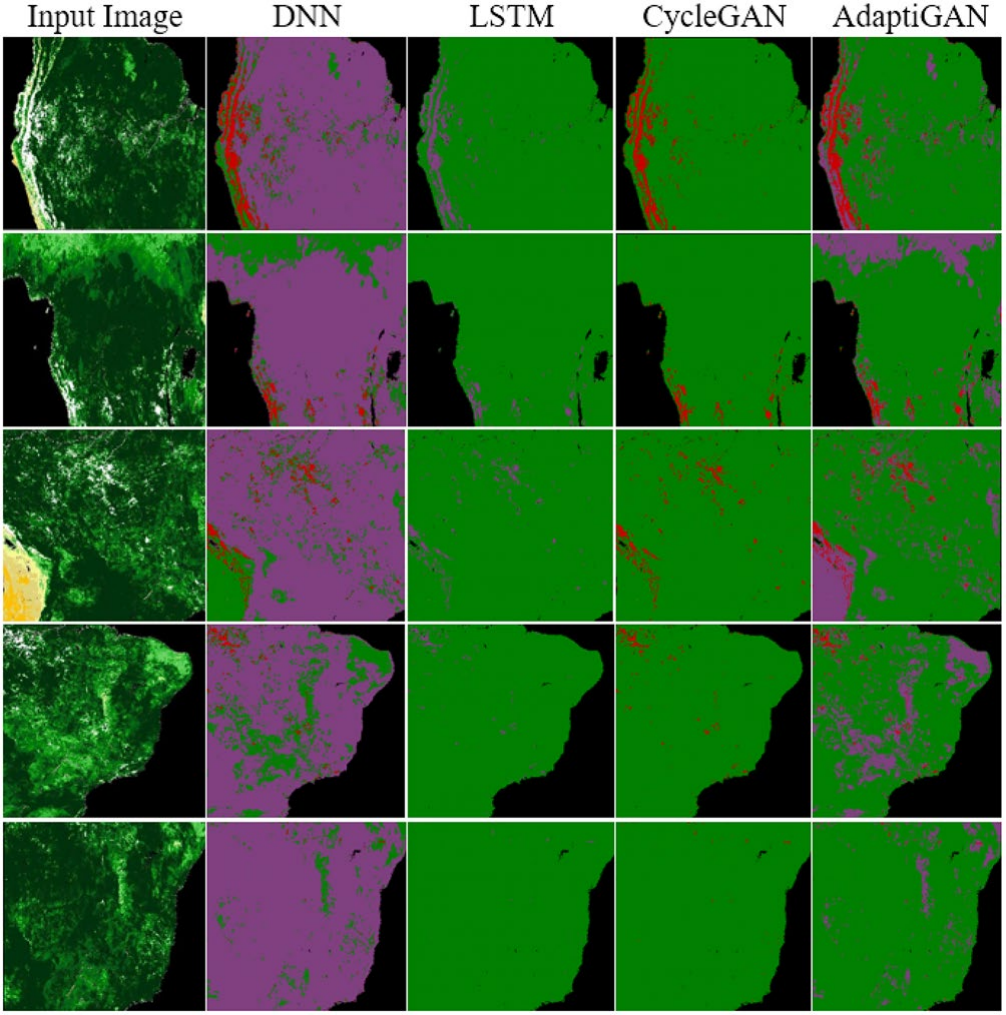
Visual comparison of various Recovery Mapping models for Amazon Region. From left to right: Original post-fire, DNN, LSTM, CycleGAN and AdaptiGAN. The maps were generated with the QGIS v.3.14 software (https://qgis.org/en/site/).

Vegetation Recovery Mapping is essential in understanding the ecological, environmental, and managerial elements of post-fire environments. This module used vegetation indices like NDVI and AdaptiGAN a deep learning-based neural network framework to provide a recovery map based on the input image. Figure 16 shows the qualitative comparison results of AdaptiGAN with other trained models, obtained for the Amazon rainforest.

The trained model’s performance was evaluated using evaluation metrics like Mean Squared Error (MSE), Mean Absolute Error (MAE), Mean Squared Logarithmic Error (MSLE), Root Mean Squared Error (RMSE) and Huber loss. The obtained values are tabulated in Table 7. Unlike metrics like MSE, MAE, RMSE and MSLE which are often used for evaluation purposes, the Huber loss, also known as the smooth absolute error, is a loss function that combines the best properties of both MSE and MAE. It’s less sensitive to outliers than MSE and provides a smooth transition to MAE at zero error. Here, y is an actual value, $\widehat{y}$ is the predicted value, and $\delta$ can be considered a threshold determining when to switch from quadratic to linear behaviour. The Huber loss formula can be seen in Eq. (3).

**Table 7:** Comparison between trained models and our proposed AdaptiGAN model.

Model	MAE	MSE	MSLE	RMSE	Huber loss
DNN	0.914	90.716	0.201	9.524	0.999
LSTM	0.723	68.481	0.191	8.275	0.989
CycleGAN	0.593	49.329	0.187	7.023	0.975
AdaptiGAN	0.075	6.863	0.167	2.619	0.926

3 $$Huber\left(y,\widehat{y}\right)=\left\{\begin{array}{c}\frac{1}{2}{(y-\widehat{y})}^{2} if \left|y-\widehat{y}\right|\le \delta \\ \delta \left(\left|y-\widehat{y}\right|-\frac{1}{2}\delta \right)otherwise\end{array}\right.$$

**Figure 17:**
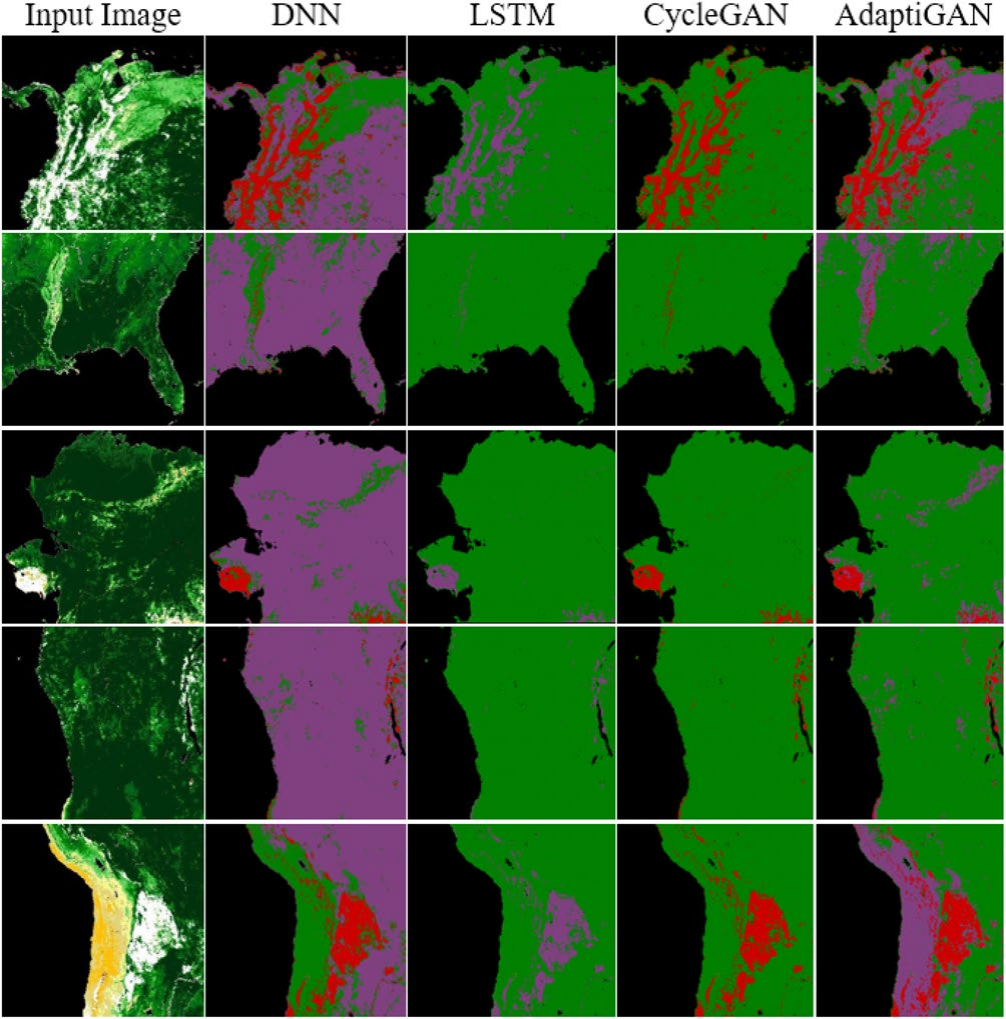
Visual comparison of various Recovery Mapping models for Knysna Region. From left to right: Original post-fire, DNN, LSTM, CycleGAN and AdaptiGAN. The maps were generated with the QGIS v.3.14 software (https://qgis.org/en/site/).

**Figure 18:**
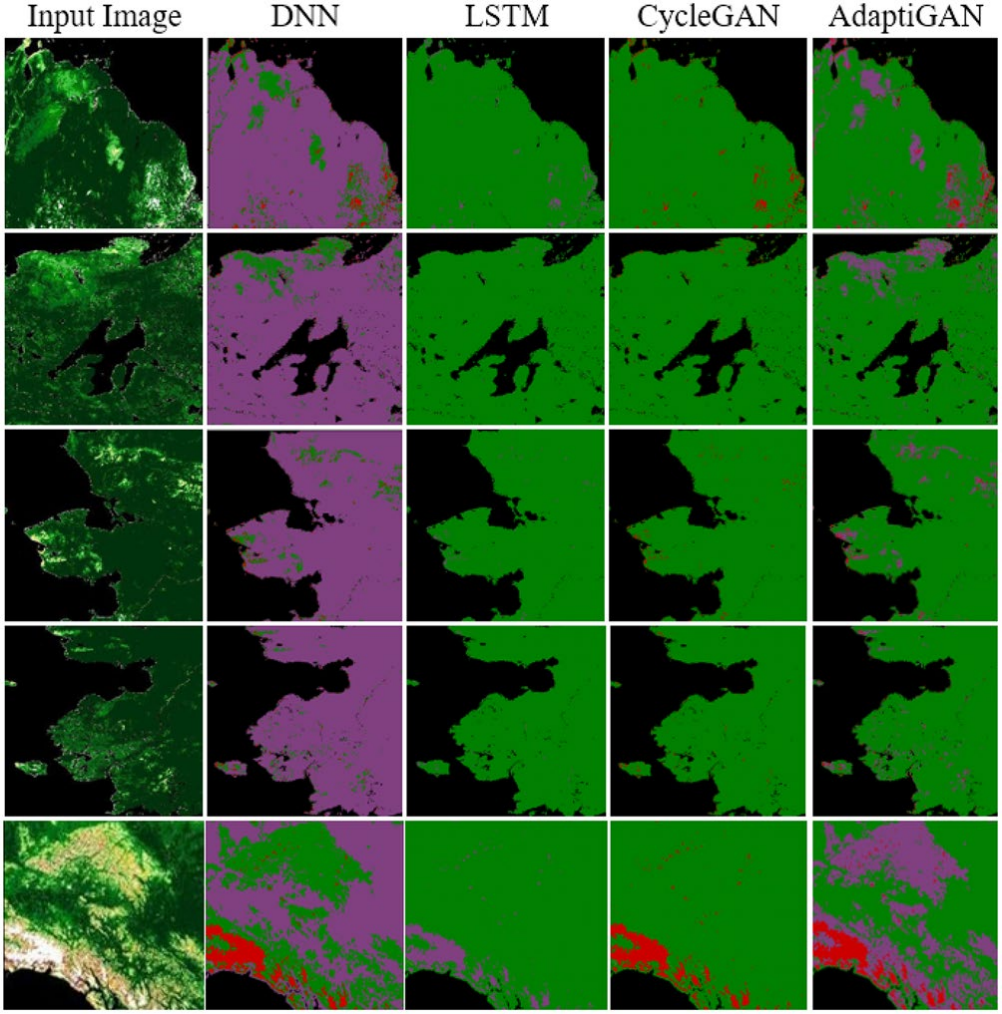
Visual comparison of various Recovery Mapping models for Alaska Region. From left to right: Original post-fire, DNN, LSTM, CycleGAN and AdaptiGAN. The maps were generated with the QGIS v.3.14 software (https://qgis.org/en/site/).

**Figure 19:**
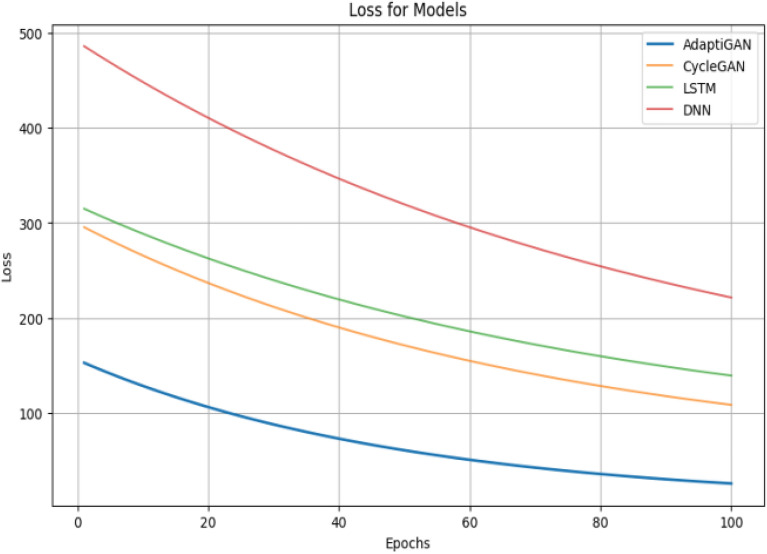
Loss Plots for pre-trained models and our proposed AdaptiGAN model.

Figure 17 shows the qualitative comparison results of AdaptiGAN with other trained models, obtained for the Knysna Region. Similarly Fig. 18 shows the comparison results obtained for our AdaptiGAN model with other trained models, obtained for the Alaska Region. Figure 19 below is a visualization of the loss variation obtained for different models along with our proposed AdaptiGAN model.

**Figure 20:**
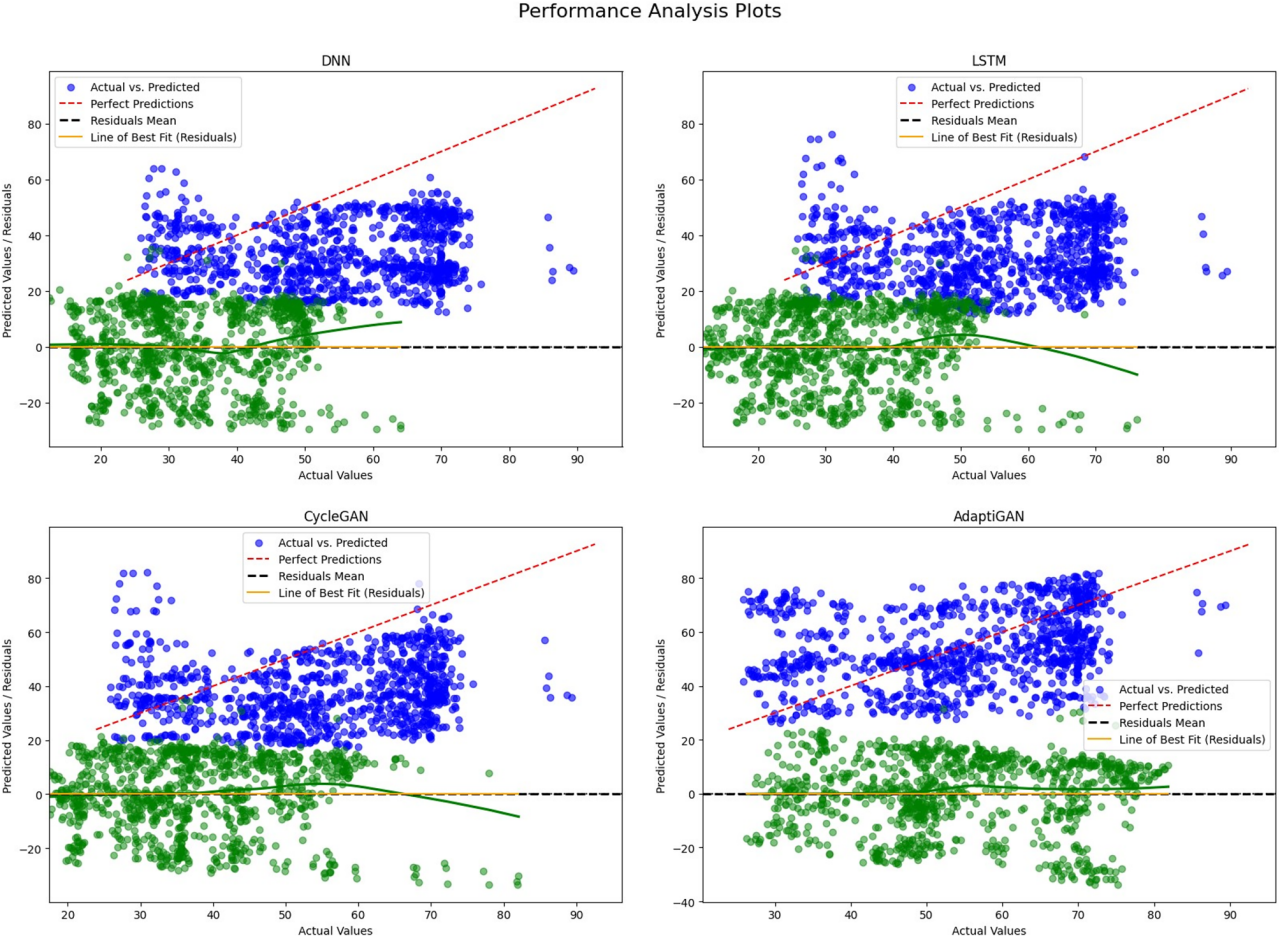
Performance Analysis of pre-trained models with proposed AdaptiGAN model.

**Figure 21:**
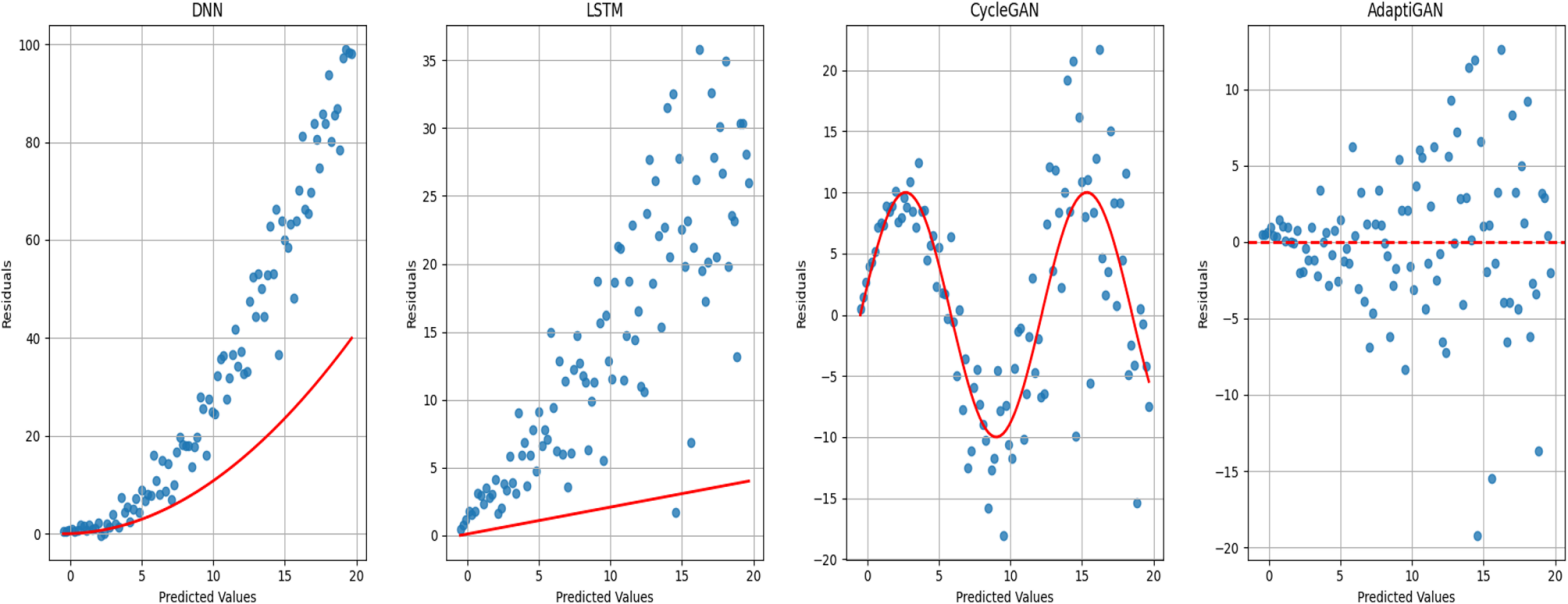
Test of Homoscedasticity Plot of pre-trained models with proposed AdaptiGAN model.

**Figure 22:**
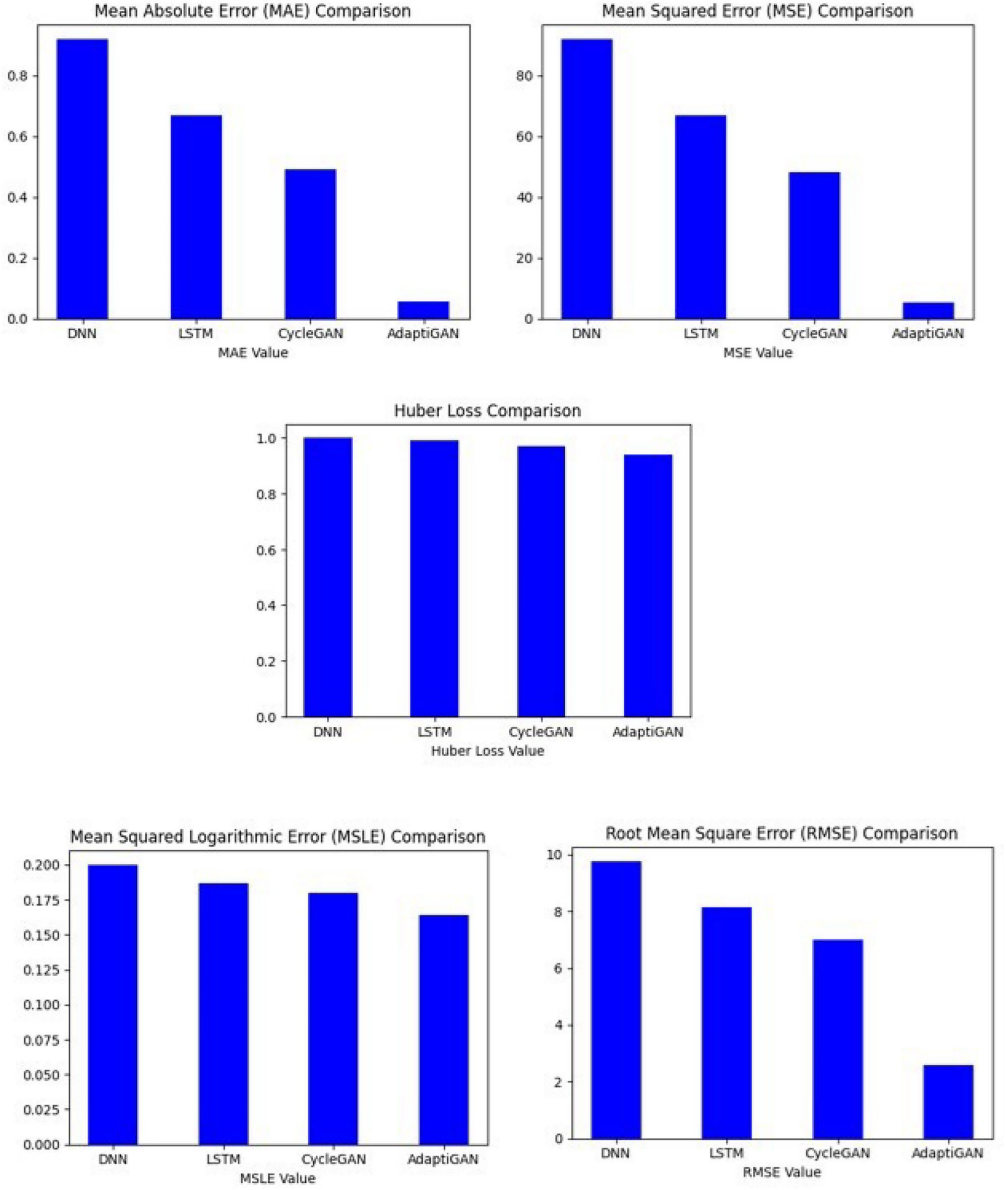
Evaluation Metrics used for comparing the performance of AdaptiGAN model with other models.

The Fig. 20 provides us with the performance-analysis plot for the pre-trained models and our AdaptiGAN Model. Figure 21 illustrates the “Test for Homoscedasticity” plot for pre-trained models compared with our AdaptiGAN model, homoscedasticity can be considered to be suitable for analysis as it provides information about whether the model fully captures the underlying patterns in the data. We can see that AdaptiGAN does not follow a clear trend while other models tend to suggest that they are heteroscedastic, which can lead to biased estimates. Figure 22 shows the plots for evaluation metrics chosen to quantify the performance of our model.

## Conclusions

We have proposed and presented an approach for analyzing the effects of a disaster like wildfires on vegetation. This approach involves vegetation change detection which is performed using an unsupervised learning algorithm called DEC along with sparse encoders for feature extraction which provides results with an impressive accuracy of 96.17%, vegetation regrowth analysis was performed using Sen’s slope estimator which provided time-series based analysis for regions affected by fire, we considered 4 creeks for this study namely: Moore, Florida, Tosher and Tonalite Creek based on which the EVI trend analysis was performed to visualize the greening and browning trend over years by using data from the MODIS dataset to analyze the EVI pattern over years along with this regrowth possibility was predicted using an Ensemble learning method called stacking which take soil data as input and provides the regrowth possibility for the region as output on whether there is a possibility for regrowth or not in that region and finally we performed vegetation recovery mapping which required collecting VIIRS data from the USGS website from which NDVI images were extracted, these NDVI images were then used for training the AdaptiGAN model. The trained model predictions were made in 3 regions chosen for this research, namely the Amazon rainforest, Knysna and Alaska regions and the corresponding recovery maps were obtained. The significant advantages of our approach include its extreme flexibility and can be used for analyzing vegetation for different regions i.e., it’s not region specific. Additionally it is efficient and can be used by organizations to investigate the effects caused by wildfires without spending much money on ground-based analysis, our approach can meet the needs posed by the real-time disaster response scenarios due to its high accuracy and speedy performance. In the future, we are planning to make predictions based on video analysis, making use of time-lapse-based satellite data and a more comprehensive technique that can effectively make note of all changes and effects caused by a wildfire by additionally incorporating the impact it had on wildlife, what all changes and precautions can be taken to prevent wildfire’s can be developed.

## Data Availability

The datasets used and/or analysed during the current study available from the corresponding author on reasonable request.
